# Guaianolide Sesquiterpenes With Significant Antiproliferative Activities From the Leaves of *Artemisia argyi*

**DOI:** 10.3389/fchem.2021.698700

**Published:** 2021-06-24

**Authors:** Wenzhuo Ming, Yi Zhang, Yiwei Sun, Guangming Bi, Jing Su, Zhutao Shao, Dali Meng

**Affiliations:** ^1^ School of Traditional Chinese Materia Medica, Shenyang Pharmaceutical University, Shenyang, China; ^2^ Chongqing Institute of Food and Drug Control, Chongqing, China

**Keywords:** *Artemisia argyi*, guaiane-type sesquiterpenoids, antiproliferative, configuration determination, antitumor

## Abstract

Four new guaiane-type sesquiterpenes, argyin H–K (1–4), and two known analogues (5 and 6) were isolated from the leaves of *Artemisia argyi* Lévl et Vant. The new compounds were characterized by the basic analysis of the spectroscopic data obtained (^1^H NMR, ^13^C NMR, HMBC, and NOESY experiments), and their absolute configurations were determined by empirical approaches, combined with the exciton chirality method and electronic circular dichroism calculations. To further understand the antitumor effects of *A. argyi*, the antiproliferative activities of these compounds against A549, MCF-7, and HepG2 cell lines were tested *in vitro* using CCK-8 assays. The results showed that these compounds had significant antiproliferative effects on MCF-7, with IC_50_ values of 15.13–18.63 μM, which were superior to that of oxaliplatin (i.e., IC_50_ 22.20 μM).

## Introduction

*Artemisia argyi* Levl. et Vant, an important species in the genus of Compositae, is distributed in China, Japan, Korea, Far East of Russia, *etc.* (Doh et al., 2016; Ozek et al., 2014). *A. argyi* often appears in people's life in various forms. As a common medicinal resource for curing eczema, diarrhea, hemostasis, and irregular menstruation in Chinese history, it has advantages of low price, easy access, wide application, and low toxicity (Li et al., 2018; Li et al., 2018; Wang et al., 2013). Pharmacological studies have shown that *A. argyi* is rich in terpenoids, flavonoids, and tannins (Zan et al., 2012; Tan and Jia, 1992; Dahae et al., 2018). *In vitro* experiments indicated that terpenoids exhibited various promising biological activities, including antibacterial, antivirus, and antitumor activities (Merfort, 2011; Zhang et al., 2005). In an effort to explore the structural diversity and biological activities of sesquiterpenes in *A. argyi*, a comprehensive phytochemical investigation was carried out, and all the compounds isolated were evaluated for their antiproliferative activities in A549, MCF-7, and HepG2 cell lines.

## Results and Discussion

### Chemistry

Compound **1** gave a molecular formula of C_20_H_28_O_7_ (HR-ESI-MS m/z 403.1728 [M + Na]^+^, calcd for 403.1733), which suggested seven unsaturation degrees. The ^1^H NMR signals at *δ* 6.04 (1H, d, *J* = 3.3 Hz) and 5.68 (1H, d, *J* = 3.3 Hz) indicated an exocyclic methylene group. In the ^13^C NMR spectrum, apart from five characteristic carbon signals for a 3-methylbutyryl group, 15 carbon resonances were observed and indicated a sesquiterpene structure.

**FIGURE 1:**
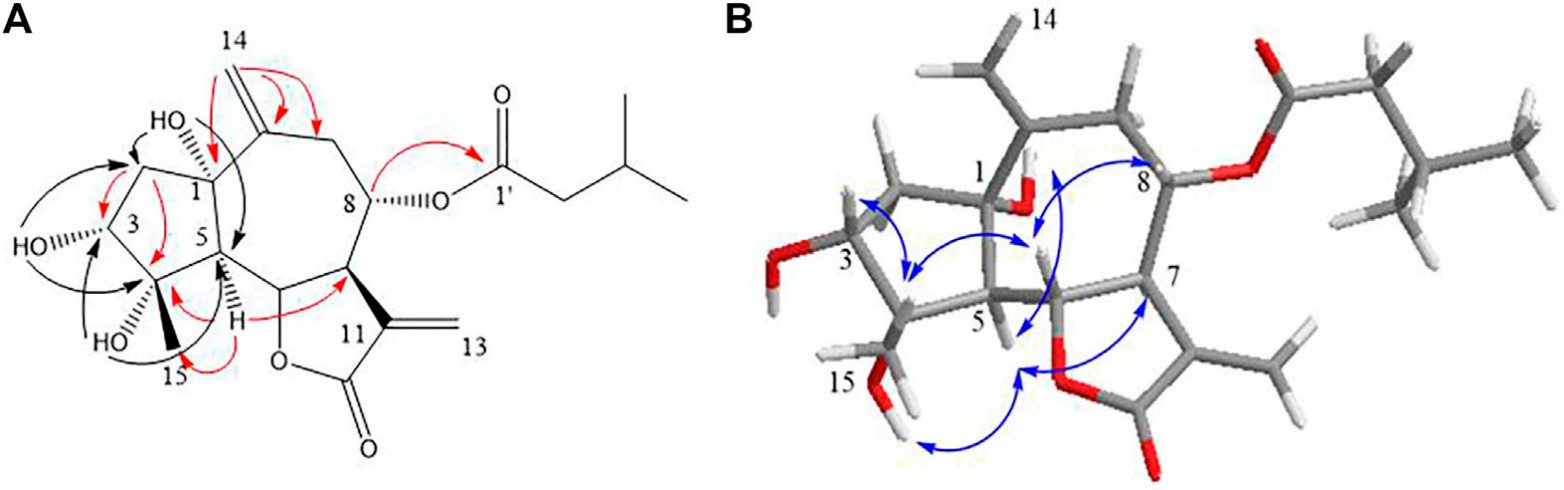
Key HMBC **(A)** and NOESY **(B)** correlations of compound **1**.

**FIGURE 2:**
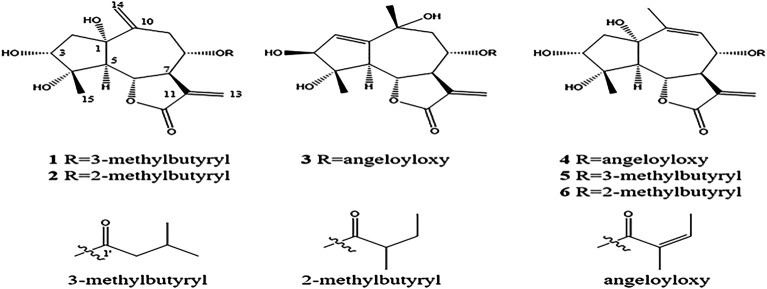
Structures of compounds **one to six** isolated from *A. argyi*.

In the HMBC spectrum (Figure 1A), the cross-peak among H-6/C-8; H-5/C-4, C-7, and C-15; H_2_-2/C-3, C-4, and C-5; and H_2_-14/C-1, C-9, and C-10 established a guaiane-type sesquiterpene skeleton with a ∆10,14 double bond. The hydroxyl substitutions at C_1_ (*δ* 5.13, 1H, s), C_3_ (*δ* 4.80, 1H, d, *J* = 4.6 Hz), and C_4_ (*δ* 4.31, 1H, s) were confirmed by the correlations of 1-OH/C-2, C-5, and C-10; 3-OH/C-2; 4-OH/C-3 and C-5, respectively. The position of 3-methylbutyryl was secured by the key HMBC correlation of H-8 (*δ*_H_ 4.85)/C-1′ (*δ*_C_ 172.1). Thus, the planar framework of **one** was established (Figure 2; Table 1).

**TABLE 1:** ^1^H NMR (DMSO-*d*_6_, 600 MHz) and ^13^C NMR (DMSO-*d*_6_, 150 MHz) spectroscopic data for compounds one to four.

Position	1		2		3		4	
	*δ*_C_	*δ*_H_ (*J* in hz)	*δ*_C_	*δ*_H_ (*J* in hz)	*δ*_C_	*δ*_H_ (*J* in hz)	*δ*_C_	*δ*_H_ (*J* in hz)
1	76.6		76.8		146.5		78.0	
2	43.7	α 1.68, br. d (13.5)	43.6	α 1.68, dd (13.5, 6.8)	128.8	5.55 d (2.1)	47.4	α 2.11, br. d (14.2, 6.3)
		β 2.00, overlapped		β 1.98, dd (13.5, 10.5)				
3	76.5	4.05, m	76.7	4.05, m	82.5	4.29 d (2.1)	78.8	3.54, m
4	80.7		80.8		84.7		79.6	
5	63.5	2.15, d (11.7)	63.6	2.16, d (11.7)	59.4	2.97 d (11.2)	61.0	2.26, d (9.6)
6	77.7	4.20, dd (11.7, 8.6)	77.7	4.22, dd (11.5, 8.7)	77.9	4.38, dd (11.2, 9.6)	75.9	4.41, t (9.6)
7	47.7	3.21, m	47.6	3.23, m	47.2	3.95, tt (9.6, 3.1)	41.8	4.24, tt (9.6, 3.2)
8	74.5	4.85, dd (10.2, 4.5)	74.4	4.87, dd (9.4, 5.0)	72.5	5.13, ddd (9.6, 5.5, 3.1)	73.0	5.31, ddd (9.6, 5.2, 1.5)
9	36.9	2.75, dd (12.1, 9.6)	36.6	2.72, dd (12.5, 9.0)	44.5	1.94, m	122.0	5.41, dd (5.2, 1.5)
		2.45, dd (12.1, 4.5)		2.42, dd (12.5, 5.0)				
10	146.0		146.0		69.7		144.6	
11	137.4		137.6		139.4		139.0	
12	169.8		169.7		169.9		169.8	
13	122.8	6.04, d (3.3)	122.5	6.05, d (2.8)	120.4	5.98, d (3.4)	121.1	6.04, d (3.0)
		5.68, d (3.3)		5.66, d (2.8)		5.40, d (3.4)		5.48, d (3.0)
14	116.2	5.03, s	116.1	5.01, s	31.0	1.33, s	25.3	1.81, s
		5.18, s		5.19, s				
15	16.5	1.02, s	16.6	1.03, s	18.2	1.12, s	23.6	1.23 s
1′	172.1		175.4		167.2		167.0	
2′	43.0	2.04, m	41.1	2.45, m	127.8		127.5	
3′	25.7	2.33, m	26.3	1.63, m	138.6	6.18, dq (1.2,7.0)	139.0	6.19, dq (7.3, 1.6)
		2.27, m		1.44, m				
4′	22.6	0.93, d, (6.6)	12.0	0.88, t, (7.0)	16.0	1.96, dq (7.3, 1.7)	16.1	1.94, dt (7.3, 1.6)
5′	22.5	0.94, d, (6.6)	17.3	1.14, d, (7.0)	20.8	1.87, t (1.7)	20.7	1.87, t (1.6)
1-OH		5.13, s		5.14, s				5.26, s
3-OH		4.80, d, (4.6)		4.81, d, (4.6)				
4-OH		4.31, s		4.33, s				4.51, s

**FIGURE 3:**
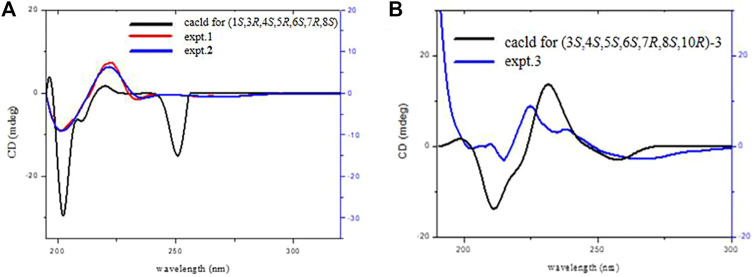
ECD spectra of compounds **1**, **2 (A)**, and **3 (B)** (data calculated using the TDDFT method at the B3LYP/6–31 + g(d,*p*) level.).

The *trans* disposition of H-5 and H-6 was deduced from the large vicinal coupling constants (*J*_5,6_ = 11.6 Hz) (Wang et al., 2014). The strong NOE correlation between H-6/H_3_-15 and H-8, H_3_-15/H-3 revealed their *cis* relationship; H-5/H-7, C_1_-OH, and C_4_-OH indicated that they have co-facial orientations and assigned as *α*-oriented (Figure 1B). The overall pattern of the experimentally CD of one well matched the calculated ECD curve, which elucidated the absolute configuration of one was 1*S*, 3*R*, 4*S*, 5*R*, 6*S*, 7*R*, 8*S*, and named argyin H (Figure 3A).

**FIGURE 4:**
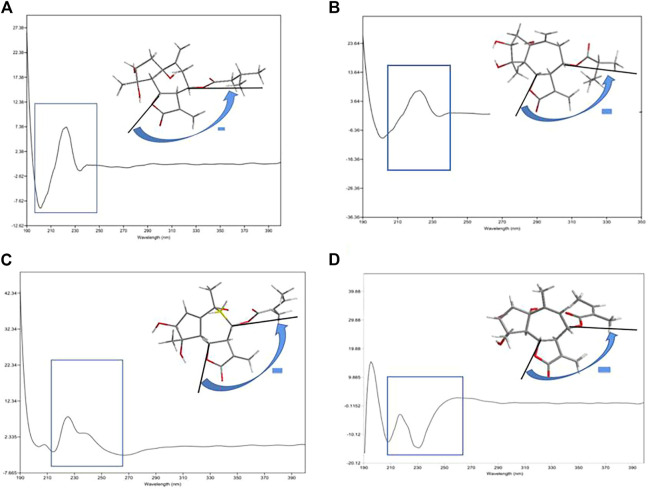
ECD exciton chirality of compounds **1 (A)**, **2 (B)**, **3 (C)**, and **4 (D)**.

The exciton chirality method in CD spectra is a very useful method to determine the absolute configuration of organic molecules. It can be used to determine not only the spatial relationship between two identical chromophores but also their absolute configuration according to the interaction of two different conjugated systems (Ying et al., 1988; Luo et al., 2011). Due to the existences of two conjugate systems in the isolated compounds, the absolute configurations could be further studied by the exciton chirality method. The ECD spectrum of one showed negative exciton chirality around the UV maximum of 234 nm; the anticlockwise array of two coupling chromophores in space (Figure 4A) and absolute configurations of two bridgehead stereogenic centers (6*S*, 8*S*) were thus determined. This result was confirmed by the unambiguous match of its experimental and calculated ECD curves.

**FIGURE 5:**
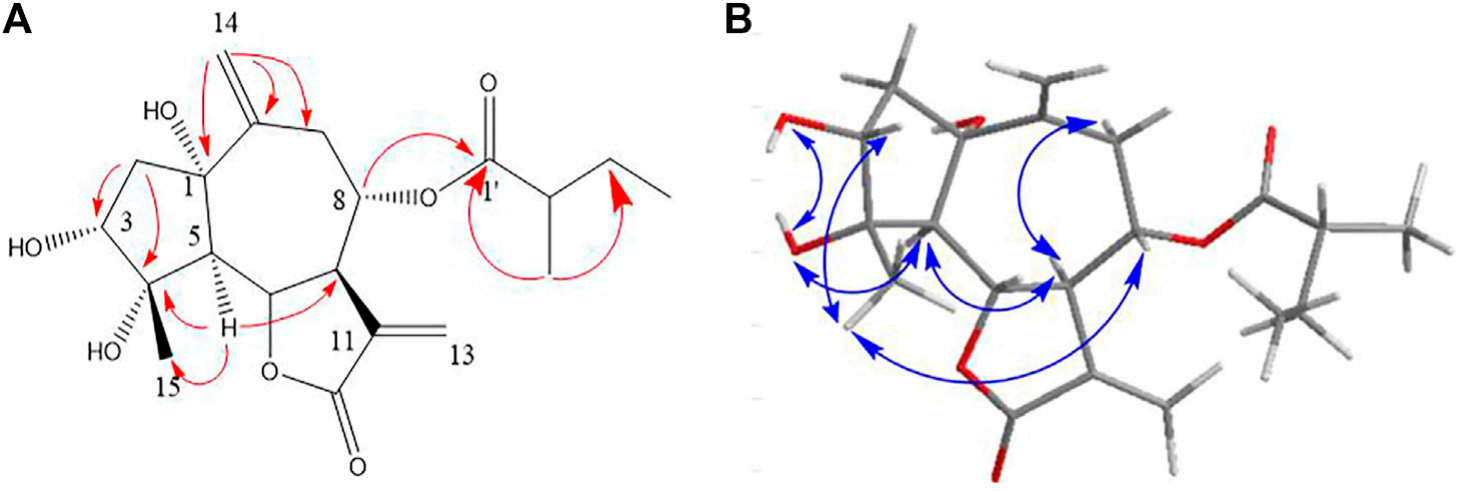
Key HMBC **(A)** and NOESY **(B)** correlations of compound **2**.

Compound **2** gave a molecular formula of C_20_H_28_O_7_ (HR-ESI-MS m/z 403.1730 [M + Na]^+^, calcd for 403.1733), which also suggested seven unsaturation degrees. Apart from the resonances attributed to 2-methylbutyryloxy (*δ*_C_ 175.4, 41.1, 26.3, 12.0, and 17.3) instead of 3-methylbutyryloxy group, ^1^H NMR, ^13^C NMR, and HMBC spectra were almost similar to those of **1**, and the planar framework of **two** was also established by 1D NMR along with HMBC data (Figures 2, 5; Table 1).

The large vicinal coupling constants of H-5/6 (*J*_5,6_ = 11.5 Hz) indicated they were *trans* disposition (Wang et al., 2014). The strong NOE correlation between H-7/H-5 and H-9α, H-5/4-OH, and 1-OH revealed their *cis α*-orientation. In addition, the correlations of H_3_-15/H-3, H-6, and H-8/H-9*β* indicated the *β-*oriented of H_3_-15, H-3, and H-8 (Figure 5B). The experimental ECD curve of **two** resembled that of **1** and led to the assignment of the absolute configuration 1*S*, 3*R*, 4*S*, 5*R*, 6*S*, 7*R*, and 8*S* (Figure 3A). The negative exciton chirality around the UV maximum of 236 nm (Figure 4B) also illustrated the absolute configurations of 6*S*, 8*S*, and two was finally named argyin I.

Compound **3** gave a molecular formula of C_20_H_26_O_7_ with an HR-ESI-MS ion at m/z 401.1553 [M + Na]^+^ (calcd for 401.1576), which suggested eight unsaturation degrees. The ^1^H NMR signals at *δ* 5.98 (1H, d, *J* = 3.4 Hz) and 5.40 (1H, d, *J* = 3.4 Hz) indicated an exocyclic methylene group. The 1D NMR and HMBC data were similar to 3*α*,4*α*,10*β*-trihydroxy-8*α*-acetoxyguai-1,11 (13)-dien-6*α*,12-olide (Ahmed et al., 2004). The difference was the angeloyloxy substitution at C-8 instead of acetoxyl in the known compound (Figure 2; Table 1).

**FIGURE 6:**
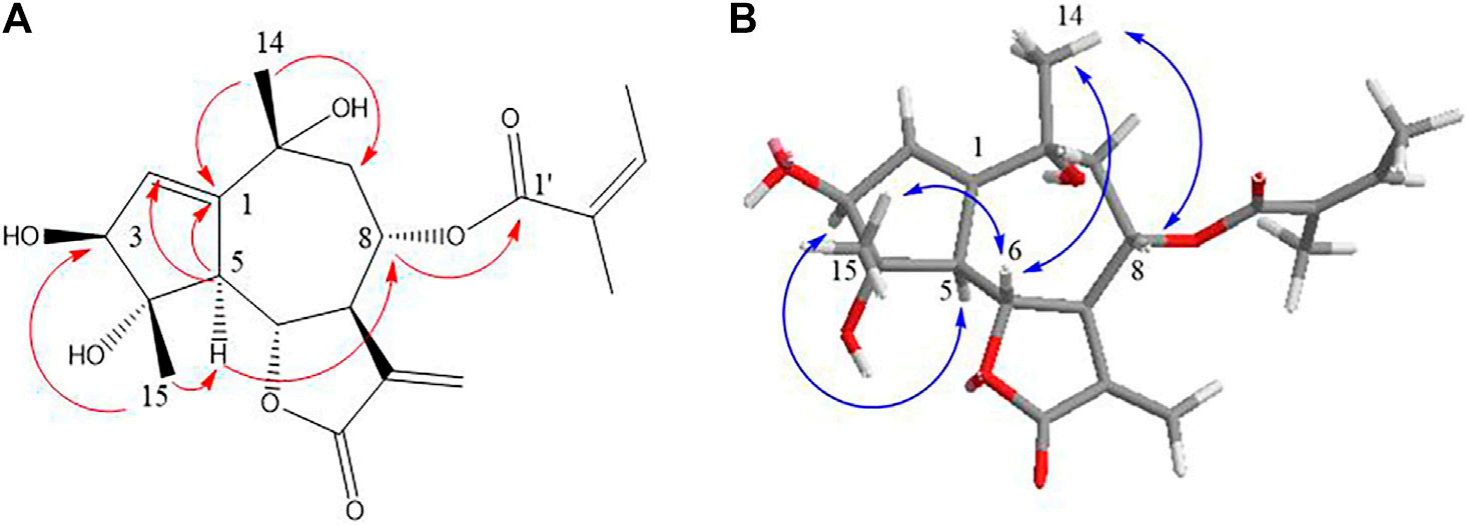
Key HMBC **(A)** and NOESY **(B)** correlations of compound **3**.

In the HMBC spectrum (Figure 6A), the cross-peak between H-5/C-1, C-2; H_3_-14/C-1 determined the location of 1,2-double bond. The HMBC correlation of H-8 (*δ*_H_ 5.13)/C-1′ (*δ*_C_ 167.2) confirmed the substituent group at C-8. *J*_5,6_ = 11.2 Hz and *J*_6,7_ = 9.6 Hz indicated that H-5, H-6, and H-7 were reciprocal *trans* oriented (Ahmed et al., 2004). The NOESY correlations (Figure 6B) between H-3/H-5 suggested that they were *α*-oriented. Additionally, H-6/H_3_-14 and H_3_-15, H_3_-14/H-8 correlations and the lack of NOE cross-peak between H-5/H_3_-15 indicated that H-6, H-8, H_3_-14, and H_3_-15 were on the same side. The negative exciton chirality of its ECD spectrum (Figure 4C) combined with the experimental and calculated ECD curves (Figure 3B) led to the assignment of the absolute configuration 3*,4,5*,6*S*,7*R*,8*S*,10*R* of **3**, which was named as argyin J.

Compound **4** had a molecular formula of C_20_H_26_O_7_ (HR-ESI-MS m/z 401.1571 [M + Na]^+^, calcd for 401.1576), which suggested eight unsaturation degrees. ^1^H NMR signals at *δ* 6.04 and 5.48 (each 1H, d, *J* = 3.0 Hz) indicated the presence of an exocyclic methylene group. In ^13^C NMR, apart from five characteristic carbon signals (angeloyloxy group) at C_8_, other 15 carbon resonances were found to be similar to those of the previously published compound (Reinhardt et al., 2019), argyinolide N (**5**) and argyinolide M (**6**), indicating their similar structures (Figure 2; Table 1).

**FIGURE 7:**
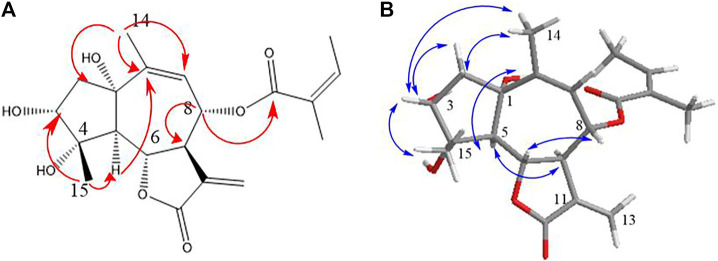
Key HMBC **(A)** and NOESY **(B)** correlations of compound **4**.

The HMBC cross-peak among H-6/C-4, C-5, and C-8; H_3_-15/C-3 and C-5; and H_3_-14/C-1, C-9, and C-10 confirmed the above planar structure deduction. H-8 (*δ*_H_ 5.31)/C-1′ (*δ*_C_ 167.0) revealed the presence of the angeloyloxy group at C-8 (Figure 7A).

The relative configuration of H-5 and H-6 was assigned to be *α*-, *β*-oriented based on their coupling constants of *J*_5,6_ = 9.6 Hz (Reinhardt et al., 2019). H-7 and OH-1 were *α*-oriented owing to the NOESY correlations of OH-1/H-5 and H-5/H-7, while H-6/H-8 correlation indicated the *α*-orientation for the 8-angeloyloxy group. Besides, H_3_-14/H-2*β* and H-3, H-3/H_3_-15 suggested H-3 and H_3_-15 were *β*-oriented (Figure 7B). In addition, the consistency of the experimental CD curve of **4** at 196 nm (+), 209 nm (−), 218 (+), and 232 nm (−) with those of argyinolide M and argyinolide N were reported previously (Reinhardt et al., 2019). Around the UV maximum of 232 nm, the ECD spectrum of **4** showed negative exciton chirality, the two carbon atoms (6, 8) were anticlockwise array, and the absolute configurations of these two carbon atoms (6*S*, 8*S*) were thus determined (Figure 7D). This result was confirmed by the match of its experimental and calculated ECD curves. Thus, **4** was established as (1*S*,3*R*,4*S*,5*R*,6*S*,7*R*,8*S*)-8-angeloyloxy-1,3,4-trihydroxy-guai-9 (10)-en-6,12-olide and named as argyin K.

### Cytotoxic Activity

In order to test the cytotoxic activities of compounds **1–6** isolated from *A. argyi*, CCK-8 assay was used to evaluate the inhibitory effects against HepG2, A549, and MCF-7 cell lines (Table 2). These six guaianolide sesquiterpenes showed strong inhibitory effect in a dose-dependent manner against three cell lines, and the most sensitive cell line is MCF-7, in which the IC_50_ of **1**–**6** (15.13–21.62 μM) was lower than that of the positive control, oxaliplatin (22.20 μM). It can be seen that the angeloyloxy substitution could strengthen the inhibitory effects compared with others.

**TABLE 2:** Antiproliferative activities of compounds one to six against A549, HepG2, and MCF-7 cell lines.

Samples	IC_50_ values (μM)		
	A549	HepG2	MCF-7
Comp.1	24.32 ± 0.34	20.42 ± 1.28	18.63 ± 1.93
Comp. 2	22.78 ± 1.70	20.02 ± 0.83	18.59 ± 0.48
Comp. 3	17.29 ± 0.98	15.13 ± 1.56	15.13 ± 0.29
Comp. 4	22.49 ± 0.78	16.30 ± 2.90	16.30 ± 1.62
Comp. 5	23.96 ± 0.22	18.82 ± 0.68	19.33 ± 1.59
Comp. 6	19.81 ± 1.32	18.17 ± 0.12	21.62 ± 0.44
Oxaliplatin	7.22 ± 1.33	13.76 ± 0.54	22.20 ± 0.78

Each value represents the mean ± SD of three independent experiments.

## Conclusion

In our continuing investigation on *A. argyi*, four undescribed guaiane-type sesquiterpenes were isolated from the *Artemisia argyi* Lévl. et Van. The antiproliferative activities of these compounds against A549, MCF-7, and HepG2 cell lines were tested *in vitro* using CCK-8 assays. Notably, these compounds could induce more cell death than positive control (oxaliplatin). Among them, compounds **3** and **4** with angeloyloxy substitution displayed the most potent antiproliferative effects, which could offer a promising lead structure with anticancer activity.

## Experimental

### General

HPLC separation was performed on SHIMADZU LC-20AR pump and a SHIMADZU SPD-20A detector (Tokyo, Japan), using the COSMOSIL C_18_ preparative column (250 × 20 mm) and YMC-pack Prep-ODS column (250 × 20 mm). HR-ESI-MS spectra (Agilent 6200 series Q-TOF spectrometer, United States) were in the m/z mode. Column chromatography: silica gel (SiO_2_, 200–300 meshes), Sephadex LH-20 (Qingdao, China), and reversed-phase ODS (Kyoto, Japan). The 1D- and 2D-NMR were texted on a Bruker ARX-600 spectrometer (Bremen, Germany) in DMSO-*d*_*6*_ (Sigma-Aldrich Company).

### Plant Material

*Artemisia argy*i (the name *Artemisia argyi* Levl. et Vant. was recorded in Chinese Pharmacopoeia for 2015) was collected in Qizhou, Hubei Province in May (spring) 2014. Prof. Jincai Lu identified this plant as *Artemisia argyi* Lévl et Vant. A voucher specimen (No. AY-1405) was deposited in the herbarium of Shenyang Pharmaceutical University.

### Extraction and Isolation

*A. argyi* (10 kg) were extracted under the heat with 70% ethanol (3*25 L). The extraction of crude extract was as mentioned before (Sun et al., 2019). Briefly, the ethanol extracts were partitioned successively with petroleum ether (PE, 4.0 g), dichloromethane (CH_2_Cl_2_, 125.0 g), ethyl acetate (EtOAc, 60.0 g), and n-butyl alcohol (n-BuOH, 8.0 g).

The CH_2_Cl_2_ extract was chromatographed on silica gel, eluting with CH_2_Cl_2_-CH_3_OH to obtain Fr. A_1_–Fr. A_6_ (100:2 to 1:1). Combinations (Fr. A_2_ and Fr. A_3_) were then applied on reversed-phase ODS washing with aqueous MeOH (25–100%). The subfraction Fr. A_2,3_ was further separated by Sephadex LH-20 to obtain Fr. A_3-1_ to Fr. A_3-5_. Fr. A_3-2_ was isolated by HPLC (27% CH_3_CN/H_2_O) to afford compound **1** (4.2 mg) and **2** (2.2 mg). Fr.C_3-5_ was further fractionated by HPLC (32% CH_3_CN/H_2_O) to give compound **3** (2.6 mg). Fr. A_4_ was isolated by HPLC with MeOH/H_2_O (58: 42) as an eluent to afford **4** (1.8 mg), **5** (3.2 mg), and **6** (5.0 mg).

Compound 1: white powder; UV (MeOH) *λ* max (log ε) 206 (0.883) nm, ECD (MeOH) λ max (Δε): 201 (Δε −9.07), 223 (Δε +7.31), 234 (Δε −1.54); ^1^H NMR and ^13^C NMR (DMSO-*d*_6_, 600/150 MHz) data, see Table 1; HR-ES-IMS m/z 403.1728 [M + Na]^+^, calcd for 403.1733.

Compound 2: white powder; UV (MeOH) λ max (log ε) 211 (2.695) nm, ECD (MeOH) λ max (Δε): 202 (Δε −9.07), 222 (Δε +6.29), 236 (Δε −1.20); ^1^H NMR and ^13^C NMR (DMSO-*d*_6_, 600/150 MHz) data, see Table 1; HR-ES-IMS m/z 403.1730 [M + Na]^+^, calcd for 403.1733.

Compound 3: white powder; UV (MeOH) λ max (log ε) 208 (1.879) nm, ECD (MeOH) λ max (Δε): 215 (Δε −3.00), 225 (Δε +8.86), 266 (Δε −2.71); ^1^H NMR and ^13^C NMR (DMSO-*d*_6_, 600/150 MHz) data, see Table 1; HR-ES-IMS m/z 401.1553 [M + Na]^+^, calcd for 401.1576.

Compound 4: white powder; UV (MeOH) λ max (log ε) 211 (2.647) nm, ECD (MeOH) λ max (Δε): 209 (Δε −13.74), 232 (Δε −15.78); 261 (Δε +1.87); ^1^H NMR and ^13^C NMR (DMSO-*d*_6_, 600/150 MHz) data, see Table 1; HR-ES-IMS m/z 401.1571 [M + Na]^+^, calcd for 401.1576.

### Cell Proliferation Assays

The cell proliferation assays were measured using the CCK-8 method (Du et al., 2020). HepG2 (liver hepatocellular cells), MCF-7 (breast cancer cells), and A549 (lung adenocarcinoma cells) were cultured at 37°C with 5% CO_2_. HepG2 and A549 cell lines were cultured in RPMI-1640 medium (10% fetal bovine); MCF-7 were cultured in DMEM medium (10% fetal bovine). The tumor cells (10^6^ cells/ml, 96-well plate) were treated in various concentrations (6.25, 12.5, 25, 50, and 100 μM) for 24 h and measured in a microplate reader (540 nm) after adding 10 μl CCK-8 (Dalian Meilun Biotechnology Co., Ltd.). Besides, oxaliplatin was used as a positive control reagent for HepG2, MCF-7, and A549 cells.

## Data Availability

The original contributions presented in the study are included in the article/Supplementary Material; further inquiries can be directed to the corresponding author.

## References

[B1] Ahmed A. A. El-Moghazy S. A. El-Shanawany M. A. Abdel-Ghani H. F. Karchesy J. Sturtz G. (2004). Polyol Monoterpenes and Sesquiterpene Lactones from the Pacific Northwest PlantArtemisiasuksdorfii. J. Nat. Prod. 67, 1705–1710. 10.1021/np049954j 15497944

[B2] Doh E. J. Paek S. H. Lee G. Lee M. Y. Oh S. E., (2016). Application of Partial Internal Transcribed Spacer Sequences for the Discrimination of *Artemisia Capillaris* from Other Artemisia Species. Evid. Based Complement. Alternat Med. 2016, 7043436–7043602. 10.1155/2016/7043436 27313651 PMC4904105

[B3] Du K. Yang X. Li J. Meng D., (2020). Antiproliferative Diterpenoids and Acetophenone Glycoside from the Roots of euphorbia Fischeriana. Phytochemistry *.* 177, 112437. 10.1016/j.phytochem.2020.112437 32559489

[B5] Lee D. Kim C. E. Park S. Y. Kim K. O. Hiep N. T. Lee D. (2018). Protective Effect of *Artemisia Argyi* and its Flavonoid Constituents against Contrast-Induced Cytotoxicity by Iodixanol in LLC-PK1 Cells. Int. J. Mol. Sci. 19, 1387–1405. 10.3390/ijms19051387 PMC5983776 29735908

[B6] Li S. Zhou S. Yang W. Meng D., (2018). Gastro-protective Effect of Edible Plant Artemisia Argyi in Ethanol-Induced Rats via Normalizing Inflammatory Responses and Oxidative Stress. J. Ethnopharmacology 214, 207–217. 10.1016/j.jep.2017.12.023 29273436

[B7] Luo J. Wang J.-S. Wang X.-B. Luo J.-G. Kong L.-Y. (2011). Phragmalin-Type Limonoid Orthoesters from *Chukrasia Tabularis Var. Velutina* . Chem. Pharm. Bull. 59, 225–230. 10.1248/cpb.59.225 21297303

[B8] Merfort I., (2011). Perspectives on Sesquiterpene Lactones in Inflammation and Cancer. Cdt 12, 1560–1573. 10.2174/138945011798109437 21561425

[B9] Ozek G. Suleimen Y. Tabanca N. Doudkin R. Gorovoy P. G. Goger F., (2014). Chemical Diversity and Biological Activity of the Volatiles of Five Artemisia Species from Far East Russia. Rec. Nat. Prod. 3, 242–261.

[B10] Reinhardt J. K. Klemd A. M. Danton O. De Mieri M. Smieško M. Huber R. (2019). Sesquiterpene Lactones from *Artemisia Argyi*: Absolute Configuration and Immunosuppressant Activity. J. Nat. Prod. 82, 1424–1433. 10.1021/acs.jnatprod.8b00791 31181920

[B11] Sun Y. W. Ju Y. Liu C. H. Du K. C. Meng D. L., (2019). Polyhydroxyl Guaianolide Terpenoids as Potential NF‐kB Inhibitors Induced Cytotoxicity in Human Gastric Adenocarcinoma Cell Line. Bioorg. Chem. 95, 103551. 10.1016/j.bioorg.2019.103551 31911301

[B12] Tan R. Jia Z., (1992). Eudesmanolides and Other Constituents from Artemisia Argyi. Planta Med. 58, 370–372. 10.1055/s-2006-961488 17226488

[B13] Wang S. Li J. Sun J. Zeng K.-w. Cui J.-r. Jiang Y. (2013). NO Inhibitory Guaianolide-Derived Terpenoids from *Artemisia Argyi* . Fitoterapia *.* 85, 169–175. 10.1016/j.fitote.2012.12.005 23262266

[B14] Wang S. Sun J. Zeng K. Chen X. Zhou W. Zhang C. (2014). Sesquiterpenes from Artemisia Argyi: Absolute Configurations and Biological Activities. Eur. J. Org. Chem. 2014, 973–983. 10.1002/ejoc.201301445

[B15] Ying B. P. Xu R. S. Mi J. F. Han J., (1988). The Absolute Configuration of Pseudolaric Acid B. J. Chem. 1, 87–88.

[B16] Zan K. Chai X.-Y. Chen X.-Q. Wu Q. Fu Q. Zhou S.-X. (2012). Artanomadimers A-F: Six New Dimeric Guaianolides from Artemisia Anomala. Tetrahedron *.* 68, 5060–5065. 10.1016/j.tet.2012.04.046

[B17] Zhang S. Won Y.-K. Ong C.-N. Shen H.-M., (2005). Anti-Cancer Potential of Sesquiterpene Lactones: Bioactivity and Molecular Mechanisms. Cmcaca 5, 239–249. 10.2174/1568011053765976 15992352

