# Supplementary figures and images for: Guaianolide Sesquiterpenes With Significant Antiproliferative Activities From the Leaves of Artemisia argyi
Source: Front Chem. 2021 Jun 24;9:698700. doi: 10.3389/fchem.2021.698700 (PMC8263895; doi:10.3389/fchem.2021.698700)

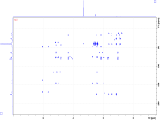

Supplement: Supplementary file 1 [file DataSheet3.ZIP › compound2/5/pdata/1/thumb.png]

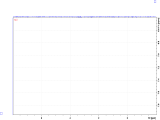

Supplement: Supplementary file 1 [file DataSheet3.ZIP › compound2/7/pdata/1/thumb.png]

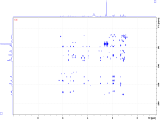

Supplement: Supplementary file 3 [file DataSheet4.ZIP › compound3/5/pdata/1/thumb.png]

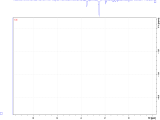

Supplement: Supplementary file 3 [file DataSheet4.ZIP › compound3/7/pdata/1/thumb.png]

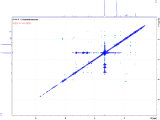

Supplement: Supplementary file 5 [file DataSheet6.ZIP › compound4 - P2/41/pdata/1/thumb.png]

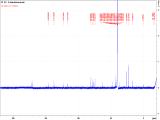

Supplement: Supplementary file 6 [file DataSheet2.ZIP › compound1/20/pdata/1/thumb.png]

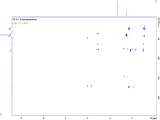

Supplement: Supplementary file 6 [file DataSheet2.ZIP › compound1/31/pdata/1/thumb.png]

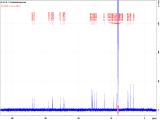

Supplement: Supplementary file 7 [file DataSheet5.ZIP › compound4 - P1/20/pdata/1/thumb.png]

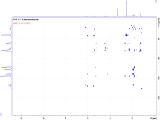

Supplement: Supplementary file 7 [file DataSheet5.ZIP › compound4 - P1/31/pdata/1/thumb.png]
