# Supplementary material for: Guaianolide Sesquiterpenes With Significant Antiproliferative Activities From the Leaves of Artemisia argyi
Source: Front Chem. 2021 Jun 24;9:698700. doi: 10.3389/fchem.2021.698700 (PMC8263895; doi:10.3389/fchem.2021.698700)
Supplement: Supplementary file 2 [file Table1.DOCX]

**Table of Contents**

**Figure S1-1** HRESIMS of compound **1**.……………….…………………................S1

**Figure S1-2** ECD of compound **1** and **2** in CH_3_OH....................................................S2

**Figure S1-3** ^1^H NMR (DMSO‑d_6_, 600 MHz) spectrum of compound **1**………....…S3

**Figure S1-4** ^13^C NMR (DMSO‑d_6_, 150 MHz) spectrum of compound **1**……….......S4

**Figure S1-5** HMBC (DMSO‑d_6_) spectrum of compound **1**.…………….….……….S5

**Figure S1-6** NOESY (DMSO‑d_6_) spectrum of compound **1**.……………….……....S6

**Figure S2-1** HRESIMS of compound **2**.……………….…………………...……….S7

**Figure S2-2** ^1^H NMR (DMSO‑d_6_, 600 MHz) spectrum of compound **2**………........S8

**Figure S2-3** ^13^C NMR (DMSO‑d_6_, 150 MHz) spectrum of compound **2**……...…....S9

**Figure S2-4** HMBC (DMSO‑d_6_) spectrum of compound **2**.………...…..…......….S10

**Figure S2-5** NOESY (DMSO‑d_6_) spectrum of compound **2**.…………….………..S11

**Figure S3-1** HRESIMS of compound **3**.……………….…………...…….………..S12

**Figure S3-2** ECD of compound **3** in CH_3_OH.………………..…………………….S13

**Figure S3-3** ^1^H NMR (DMSO‑d_6_, 600 MHz) spectrum of compound **3**………......S14

**Figure S3-4** ^13^C NMR (DMSO‑d_6_, 150 MHz) spectrum of compound **3**…...……..S15

**Figure S3-5** HMBC (DMSO‑d_6_) spectrum of compound **3**.………...….....…..…..S16

**Figure S3-6** NOESY (DMSO‑d_6_) spectrum of compound **3**.……………………...S17

**Figure S4-1** HRESIMS of compound **4**.……………….……...………….………..S18

**Figure S4-2** CD of compound **4** in CH_3_OH.………………………..……………...S19

**Figure S4-3** ^1^H NMR (DMSO‑d_6_, 600 MHz) spectrum of compound **4**………......S20

**Figure S4-4** ^13^C NMR (DMSO‑d_6_, 150 MHz) spectrum of compound **4**………….S21

**Figure S4-5** HMBC (DMSO‑d_6_) spectrum of compound **4**.……......…..….…..…..S22

**Figure S4-6** NOESY (DMSO‑d_6_) spectrum of compound **4**.………….…………..S23

**Figure S5** Docking results of compound **1** to **6**.………….………….…….…….S24


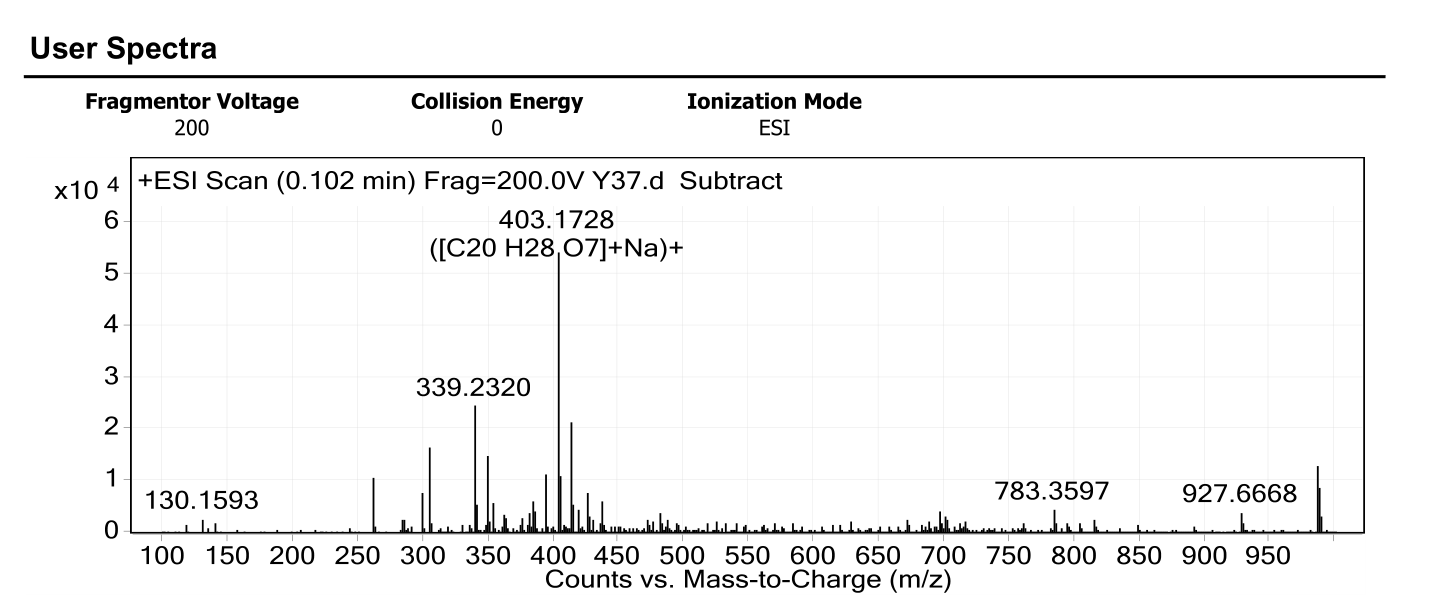


**Figure S1-1** HRESIMS of compound **1**.


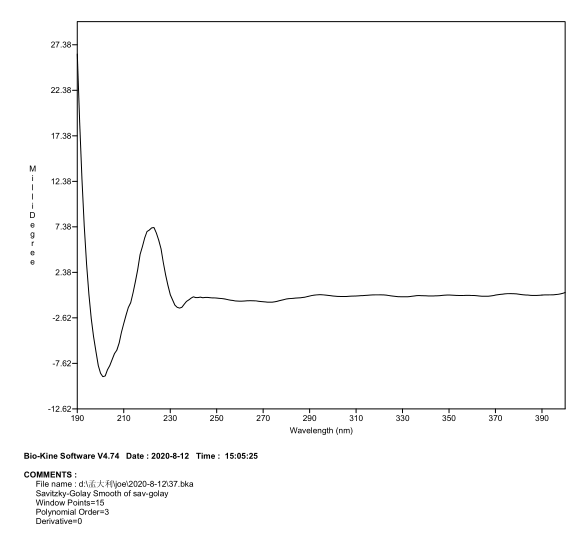


**Figure S1-2** ECD of compound **1** in CH_3_OH.


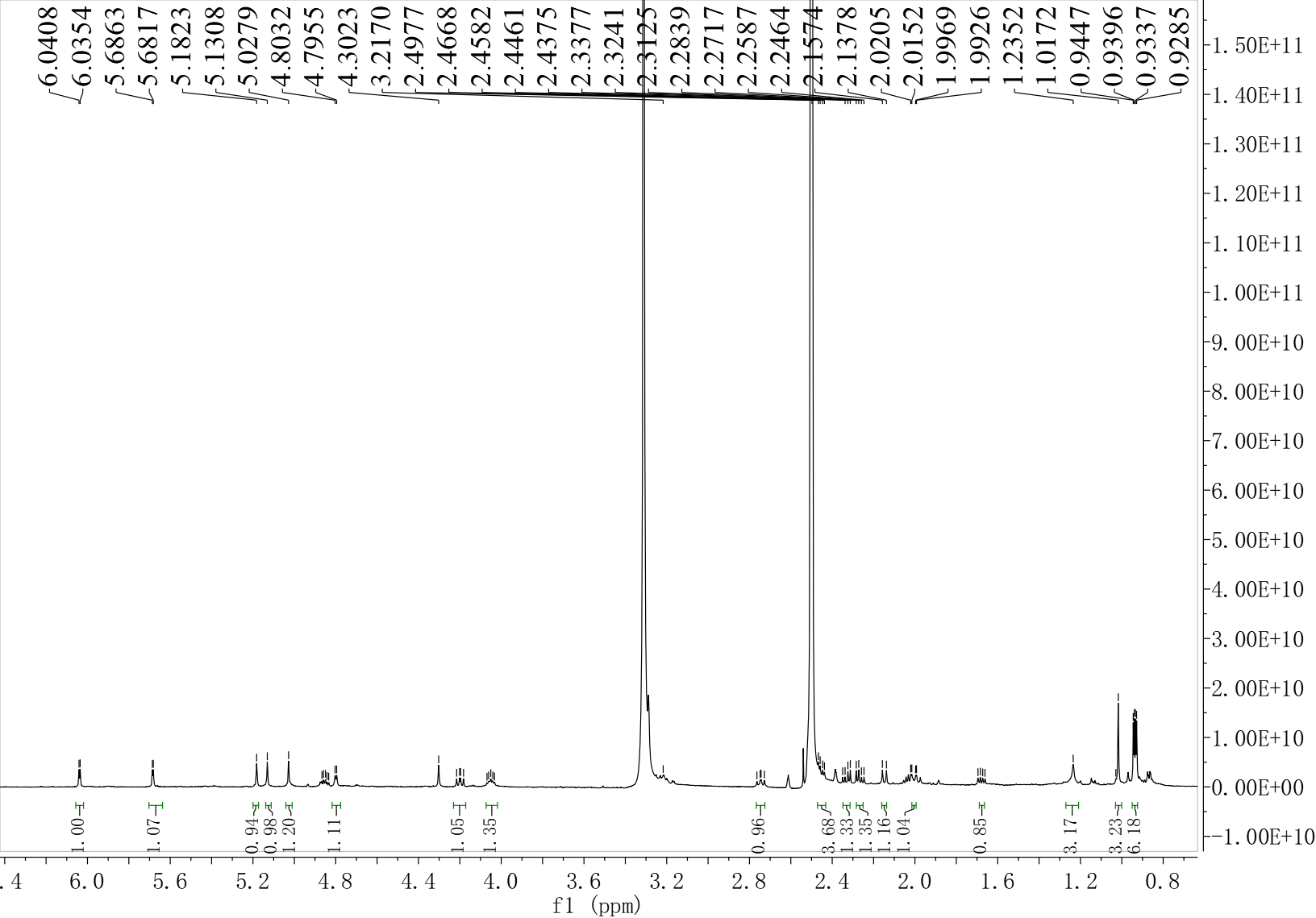


**Figure S1-3** ^1^H NMR (DMSO‑d_6_, 600 MHz) spectrum of compound **1**.


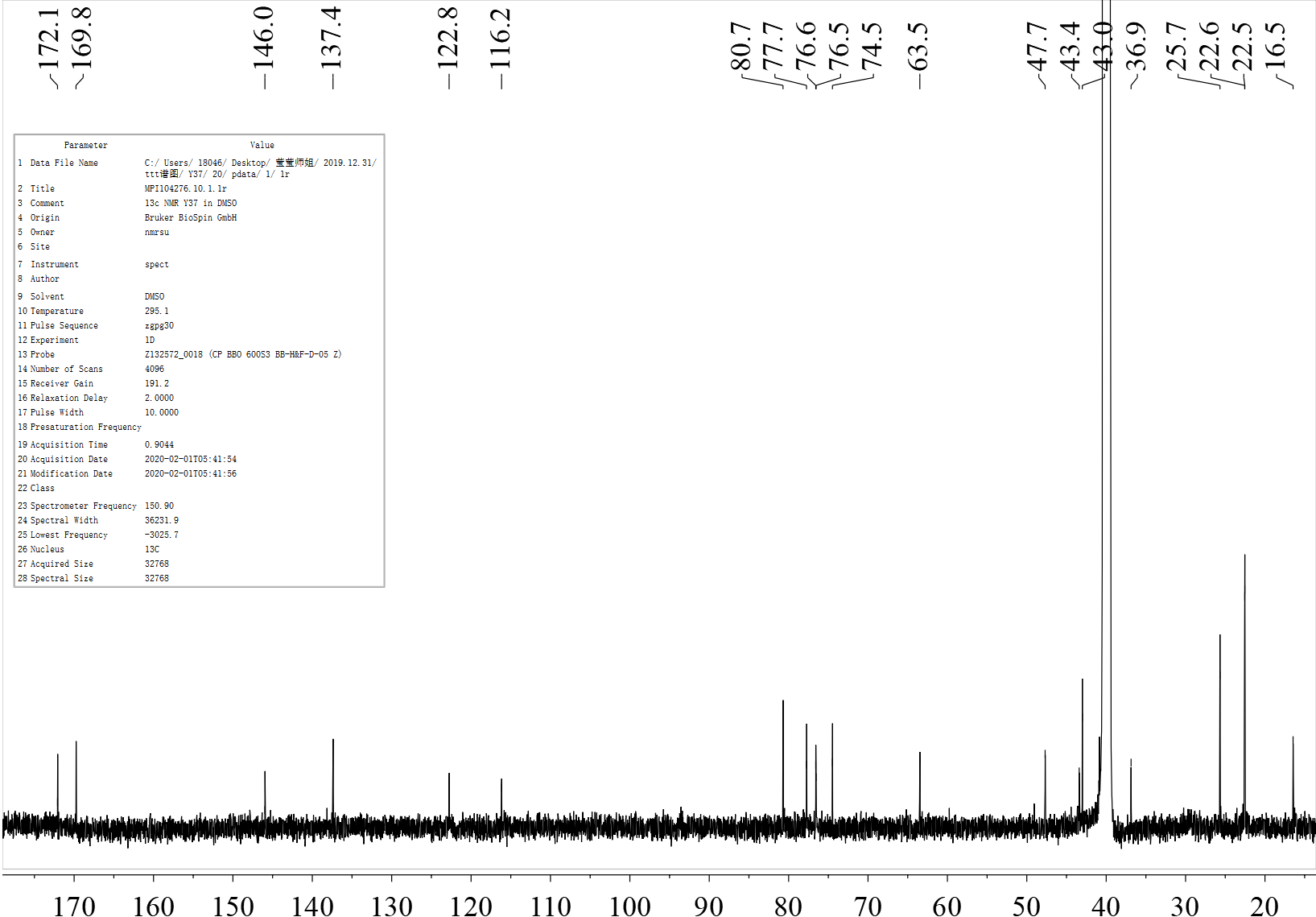


**Figure S1-4** ^13^C NMR (DMSO‑d_6_, 150 MHz) spectrum of compound **1**.


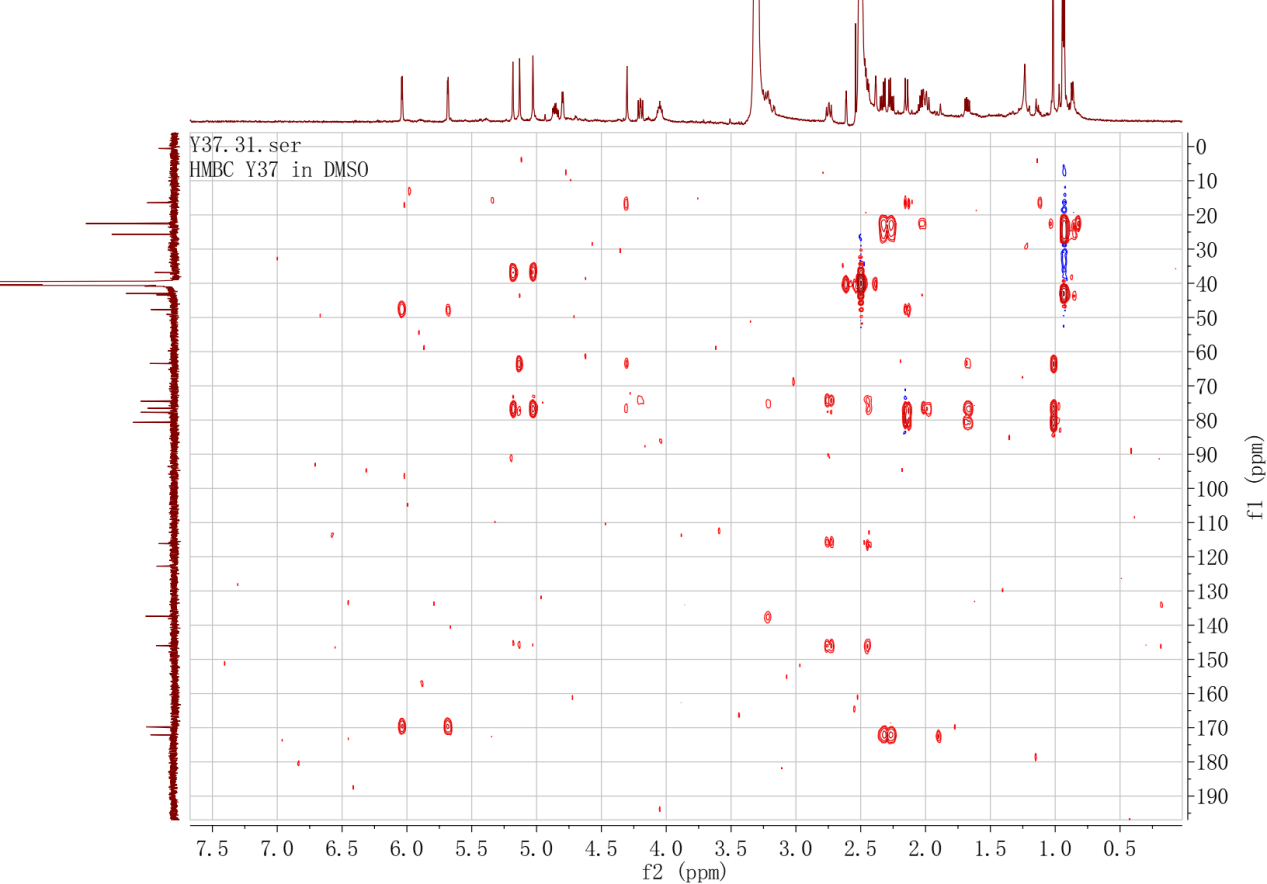


**Figure S1-5** HMBC (DMSO‑d_6_) spectrum of compound **1**.


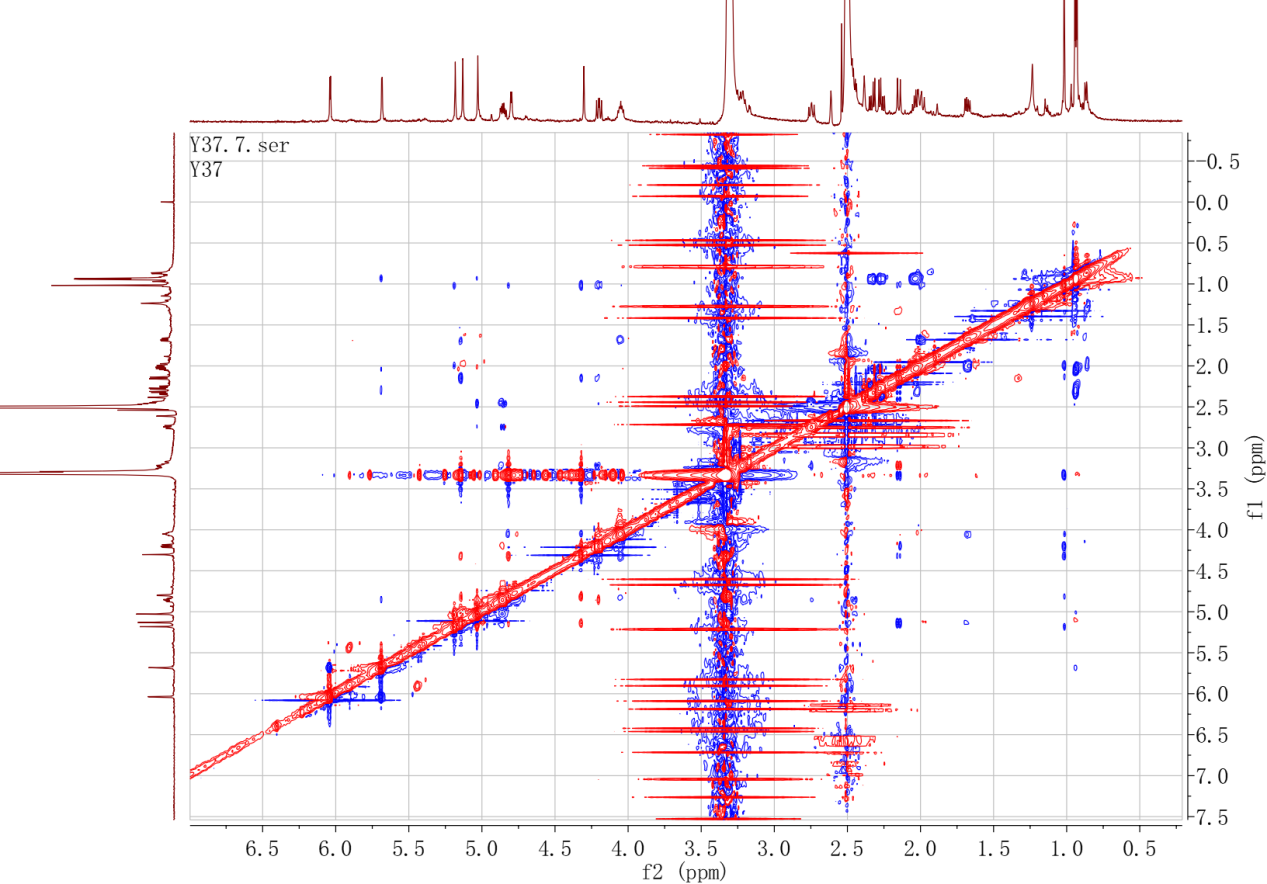


**Figure S1-6** NOESY (DMSO‑d_6_) spectrum of compound **1**.


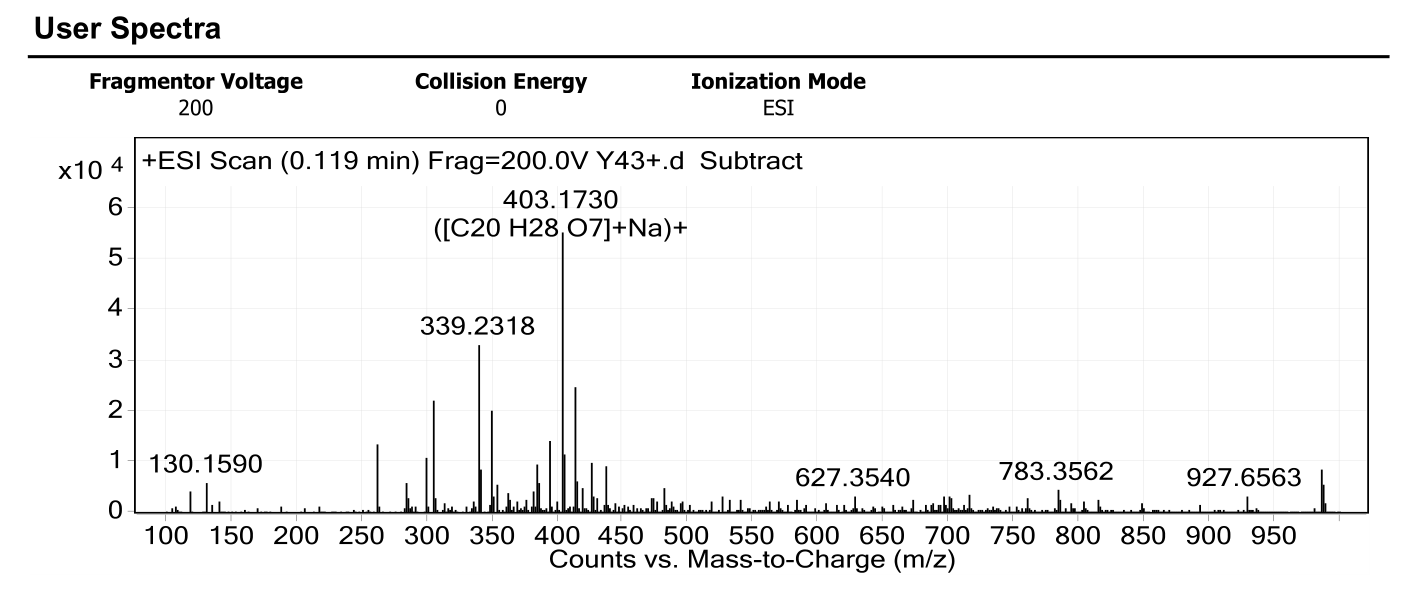


**Figure S2-1** HRESIMS of compound **2**.


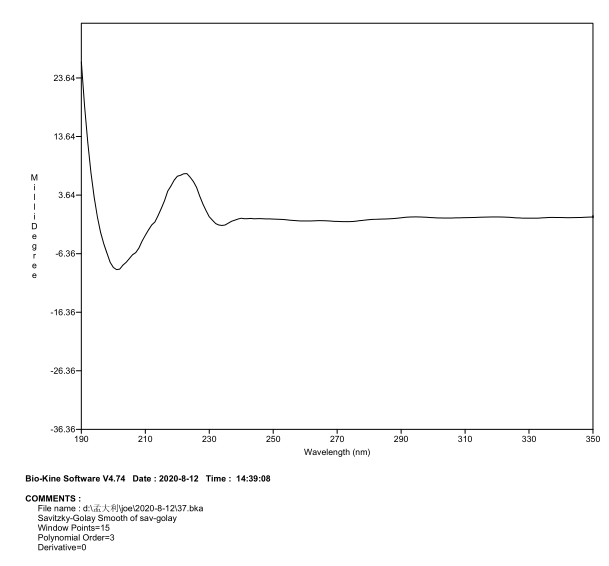


**Figure S2-2** ECD of compound **2** in CH_3_OH.


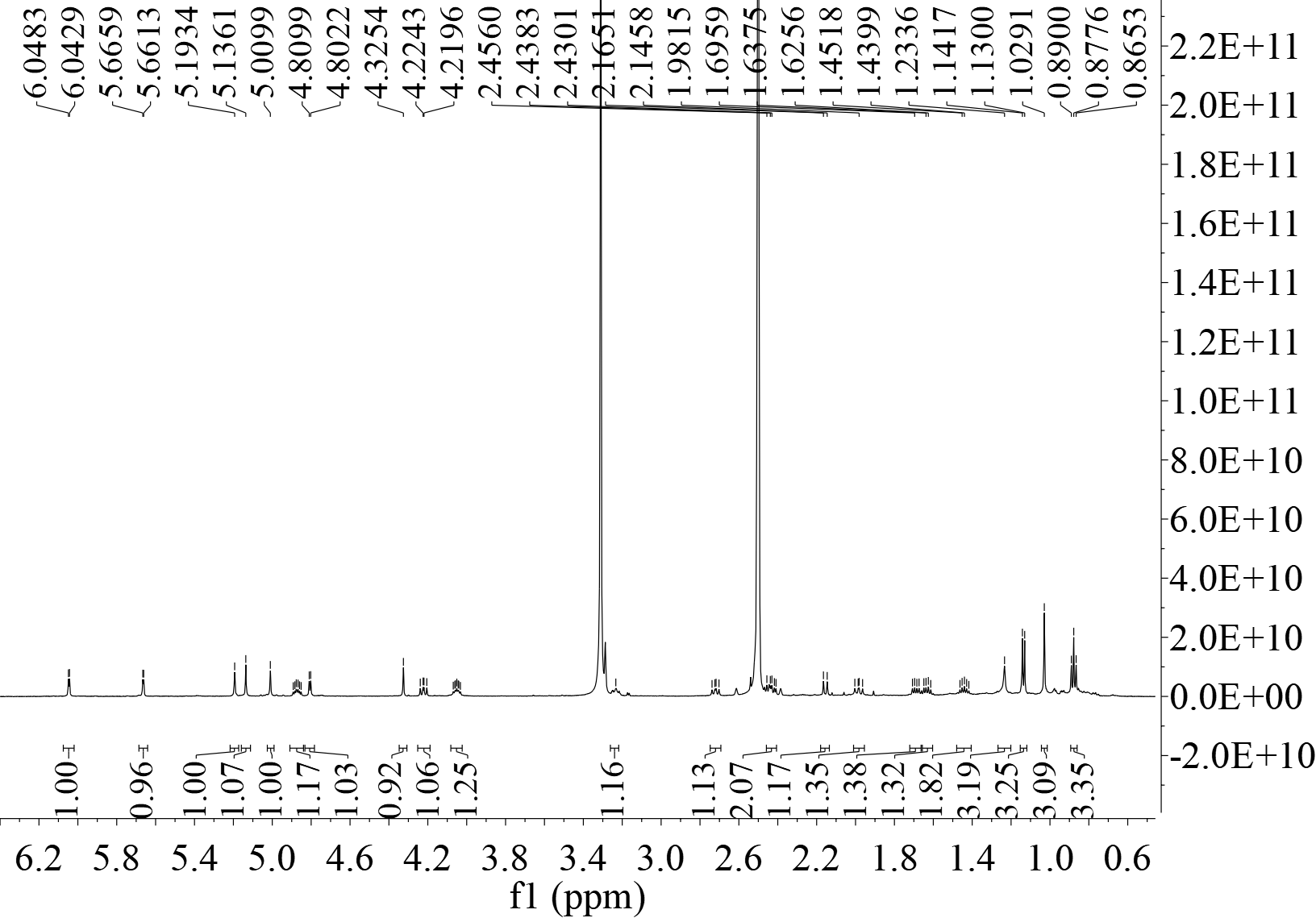


**Figure S2-3** ^1^H NMR (DMSO‑d_6_, 600 MHz) spectrum of compound **2**.


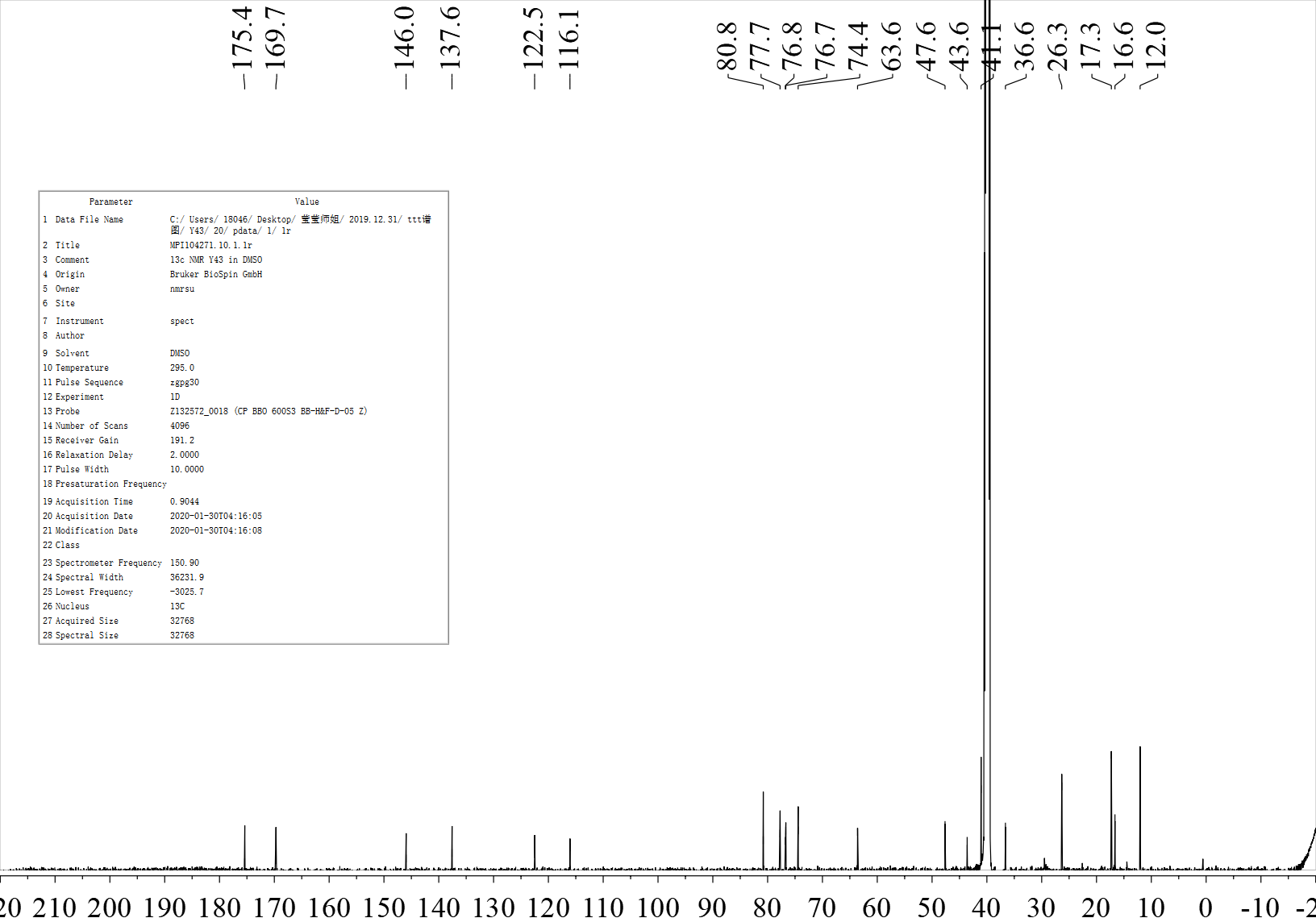


**Figure S2-4** ^13^C NMR (DMSO‑d_6_, 150 MHz) spectrum of compound **2**.


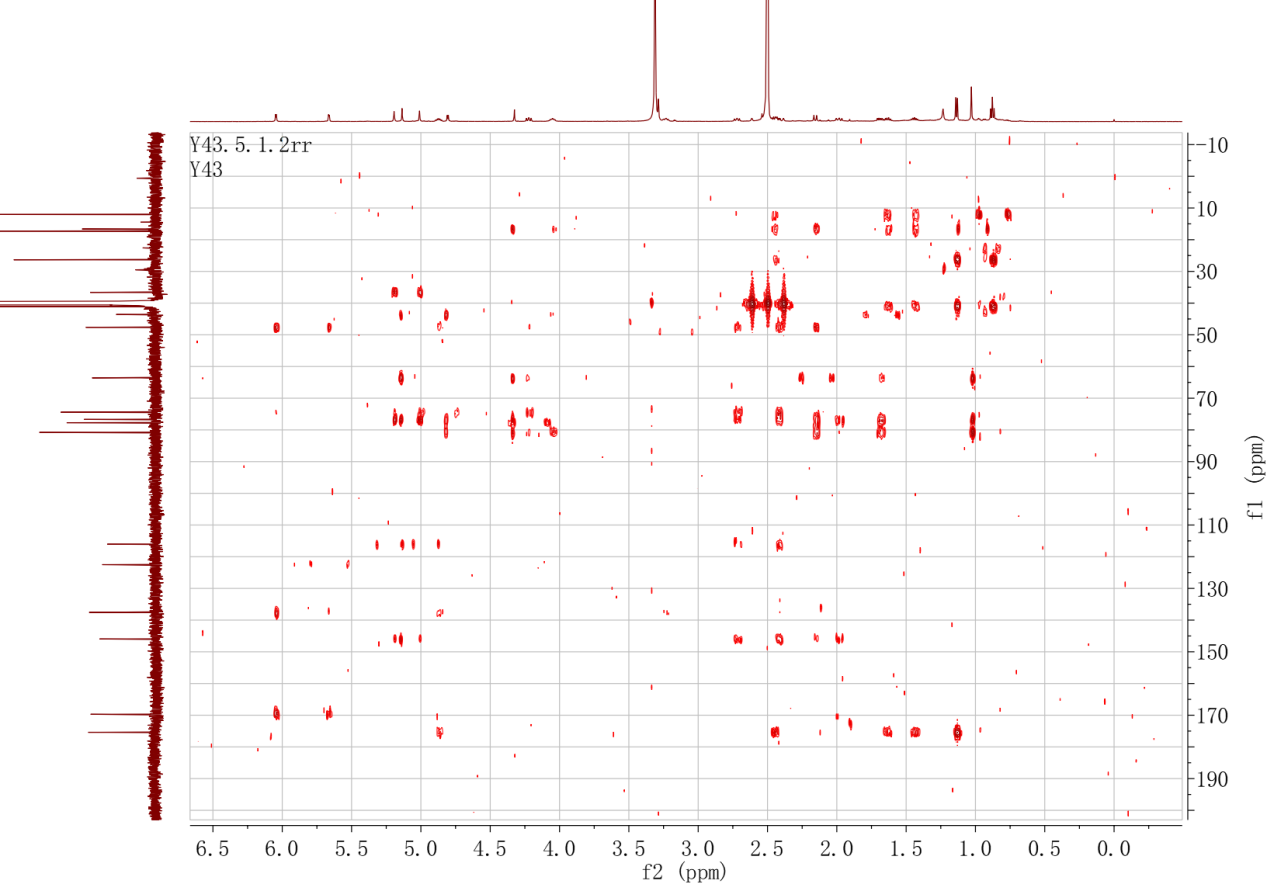


**Figure S2-5** HMBC (DMSO‑d_6_) spectrum of compound **2**.


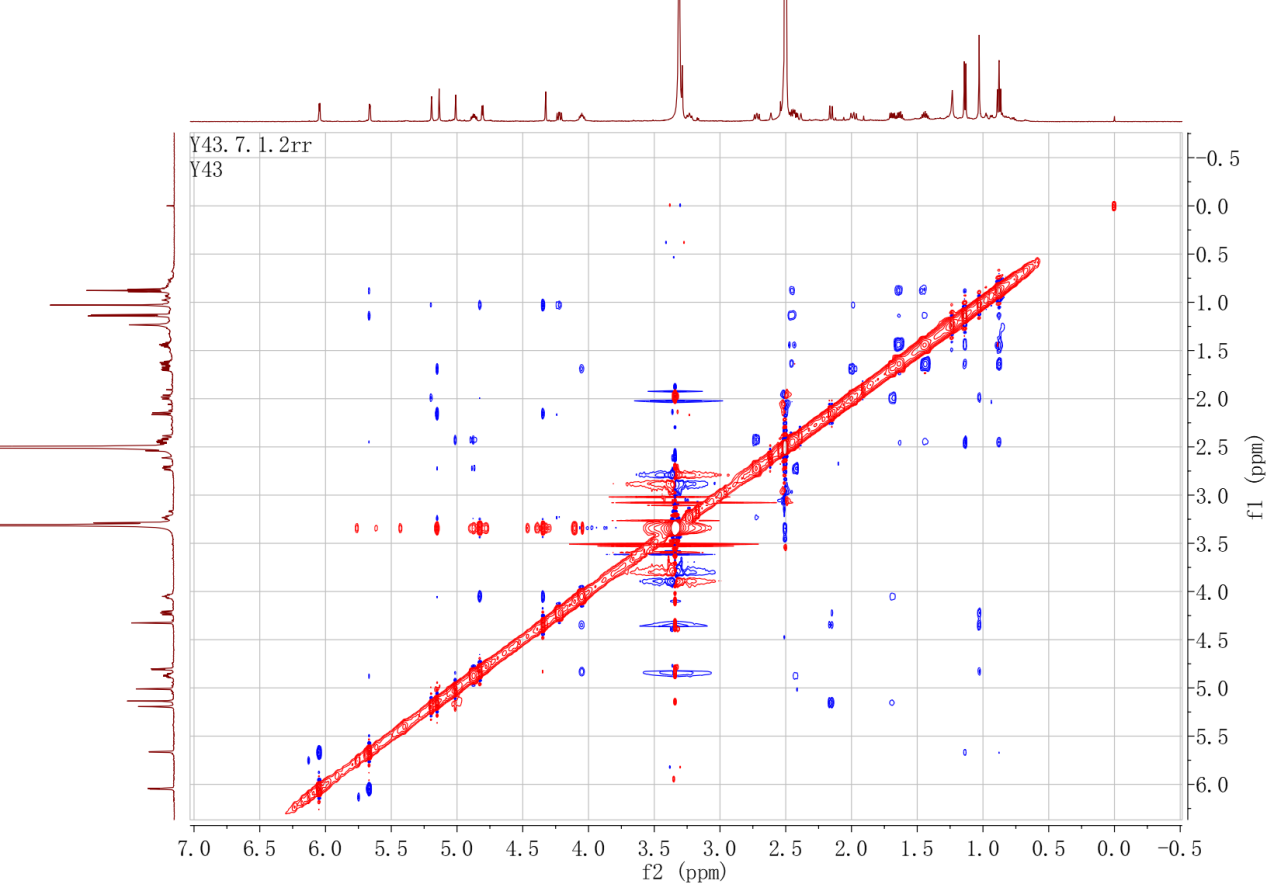


**Figure S2-6** NOESY (DMSO‑d_6_) spectrum of compound **2**.


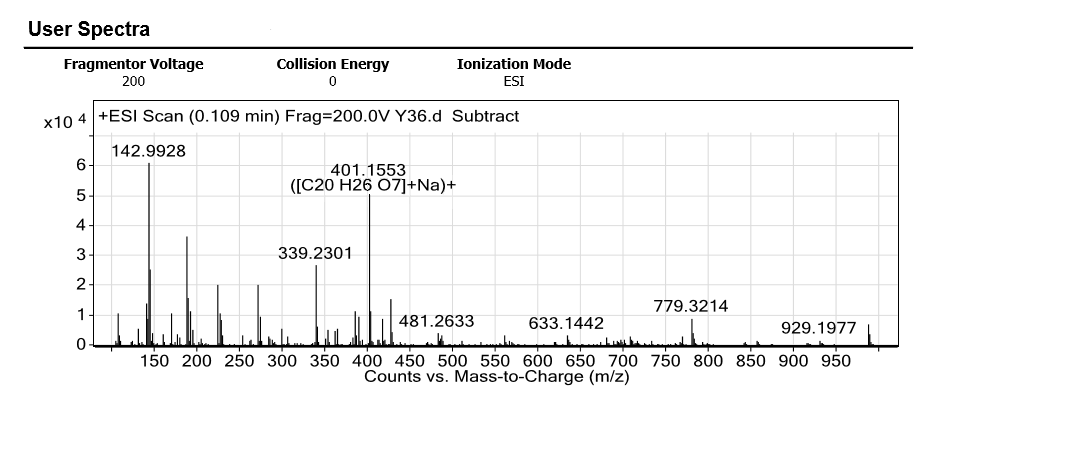


**Figure S3-1** HRESIMS of compound **3**.


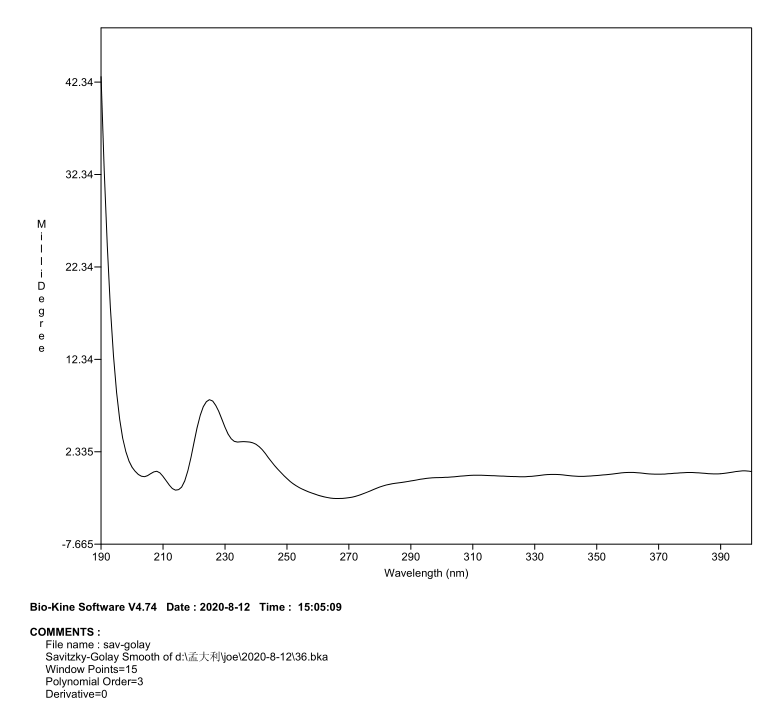


**Figure S3-2** ECD of compound **3** in CH_3_OH.


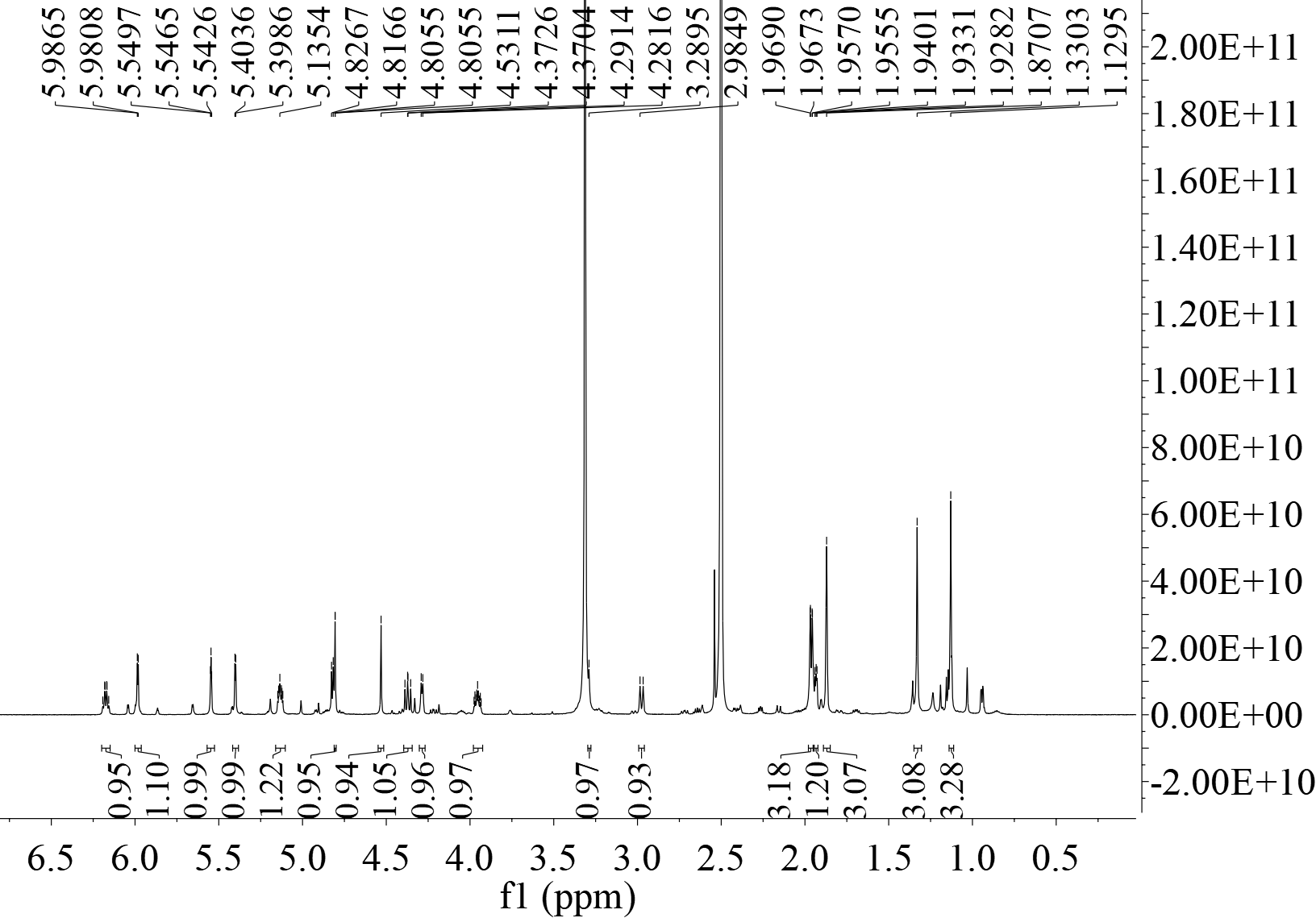


**Figure S3-3** ^1^H NMR (DMSO‑d_6_, 600 MHz) spectrum of compound **3**.


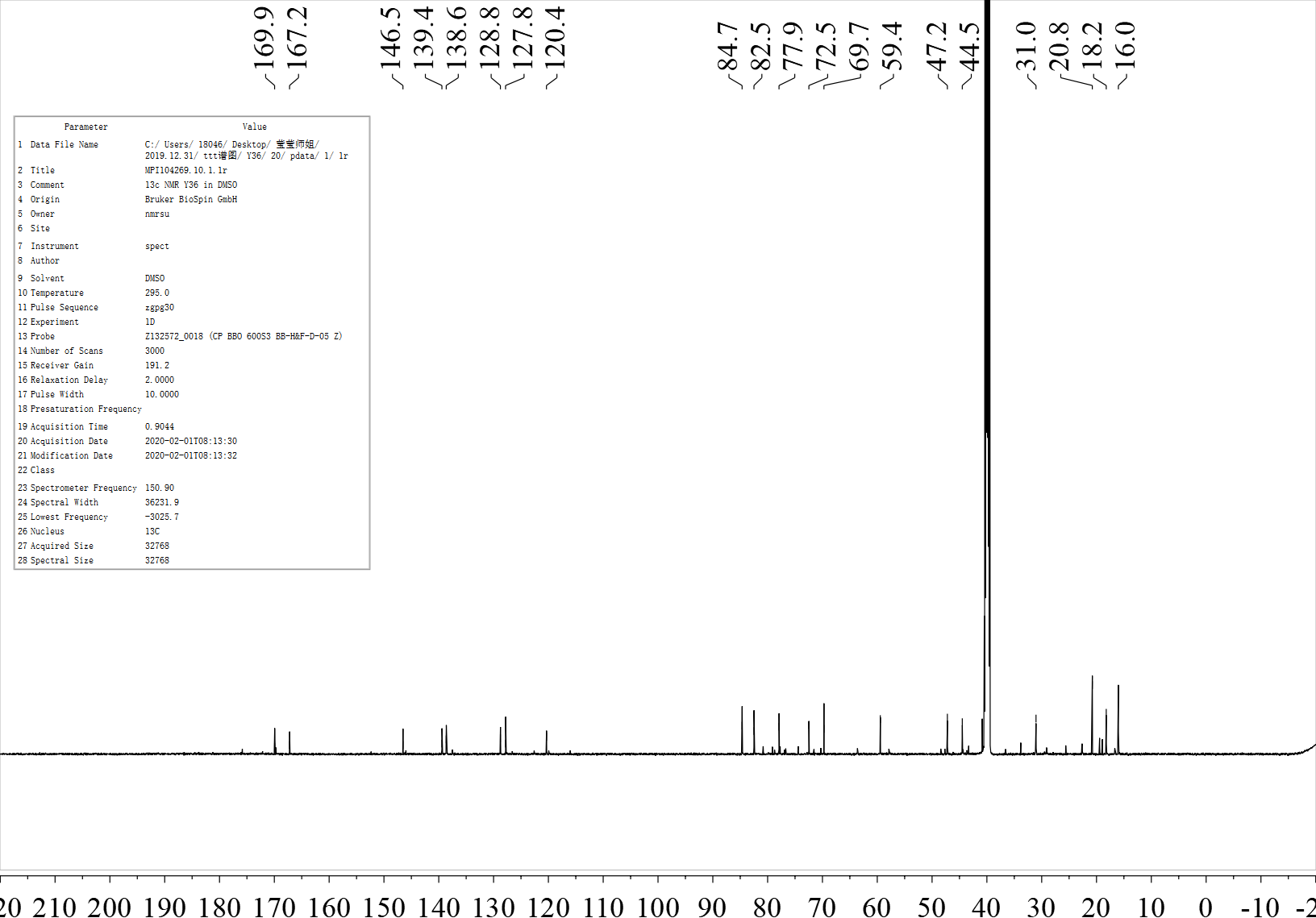


**Figure S3-4** ^13^C NMR (DMSO‑d_6_, 150 MHz) spectrum of compound **3**.


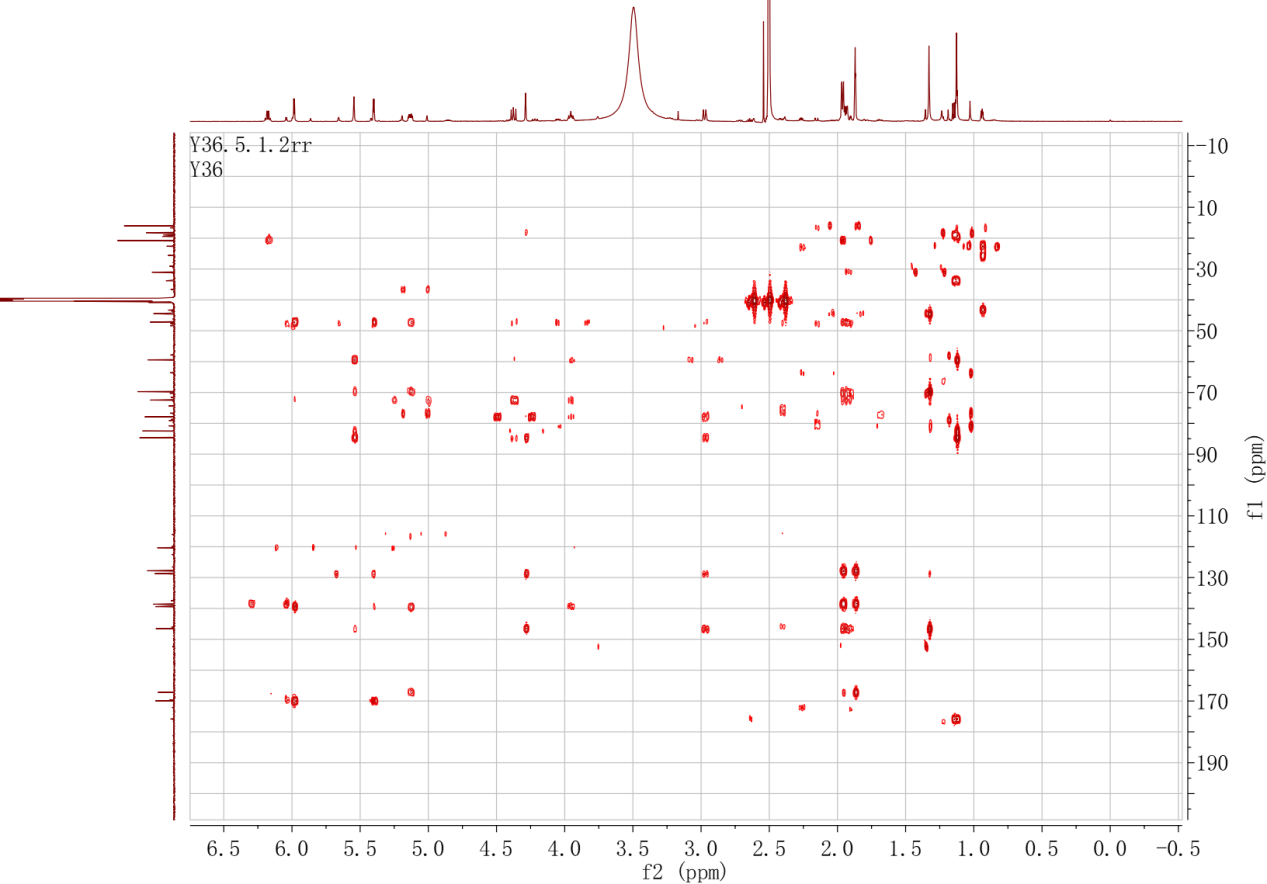


**Figure S3-5** HMBC (DMSO‑d_6_) spectrum of compound **3**.


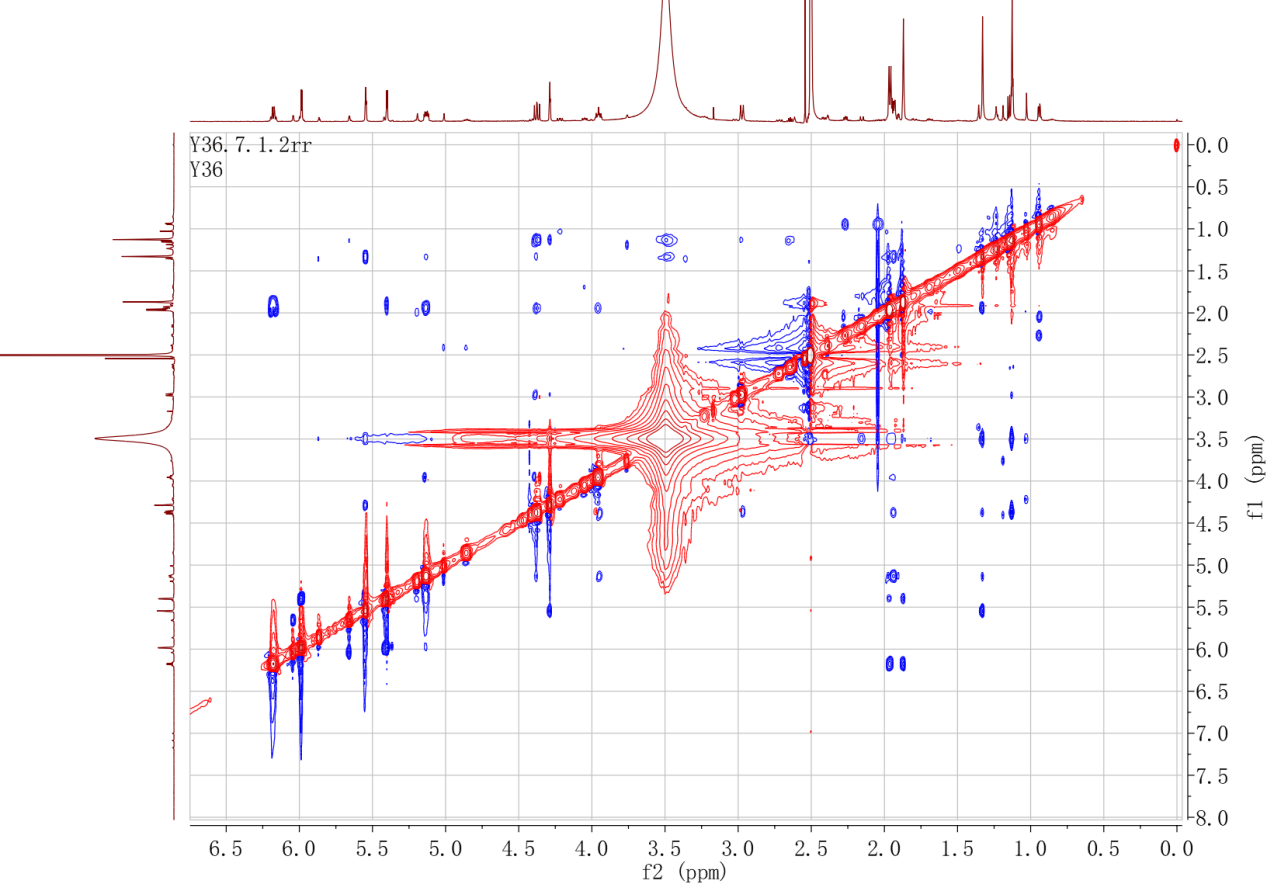


**Figure S3-6** NOESY (DMSO‑d_6_) spectrum of compound **3**.


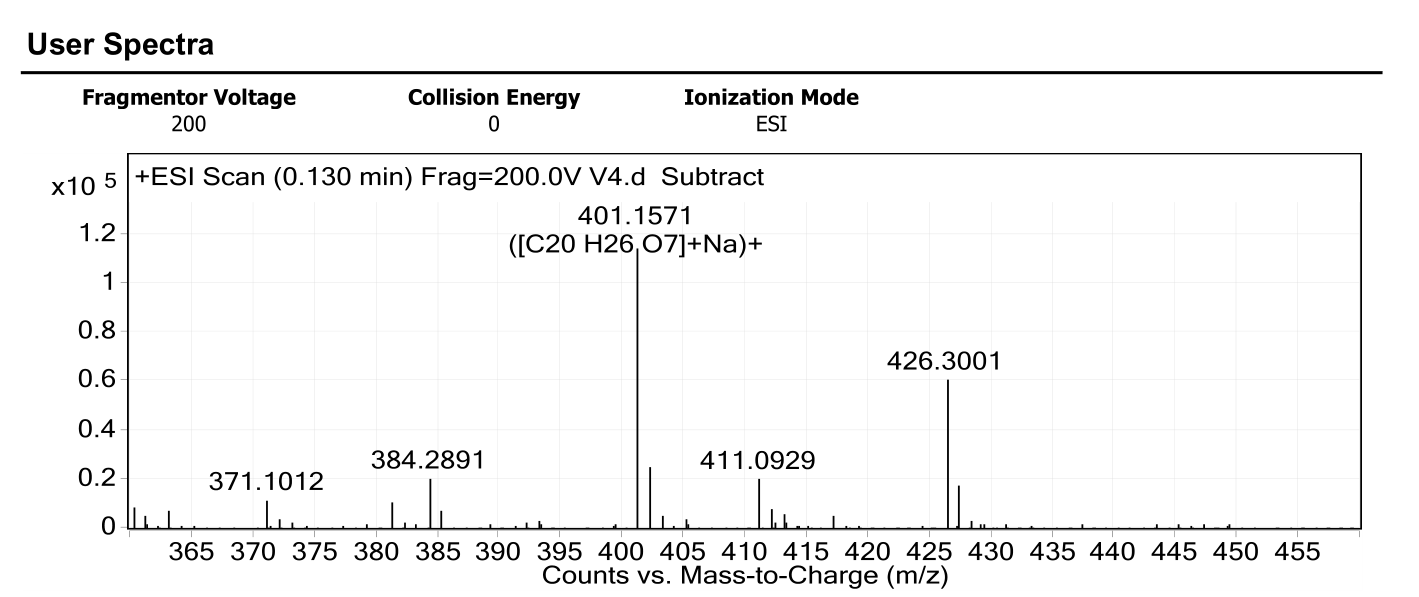


**Figure S4-1** HRESIMS of compound **4**.


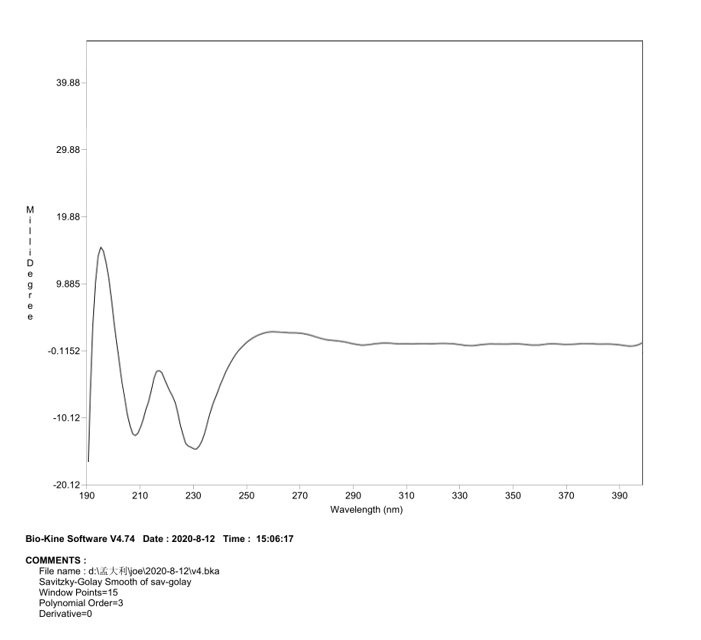


**Figure S4-2** ECD of compound **4** in CH_3_OH.


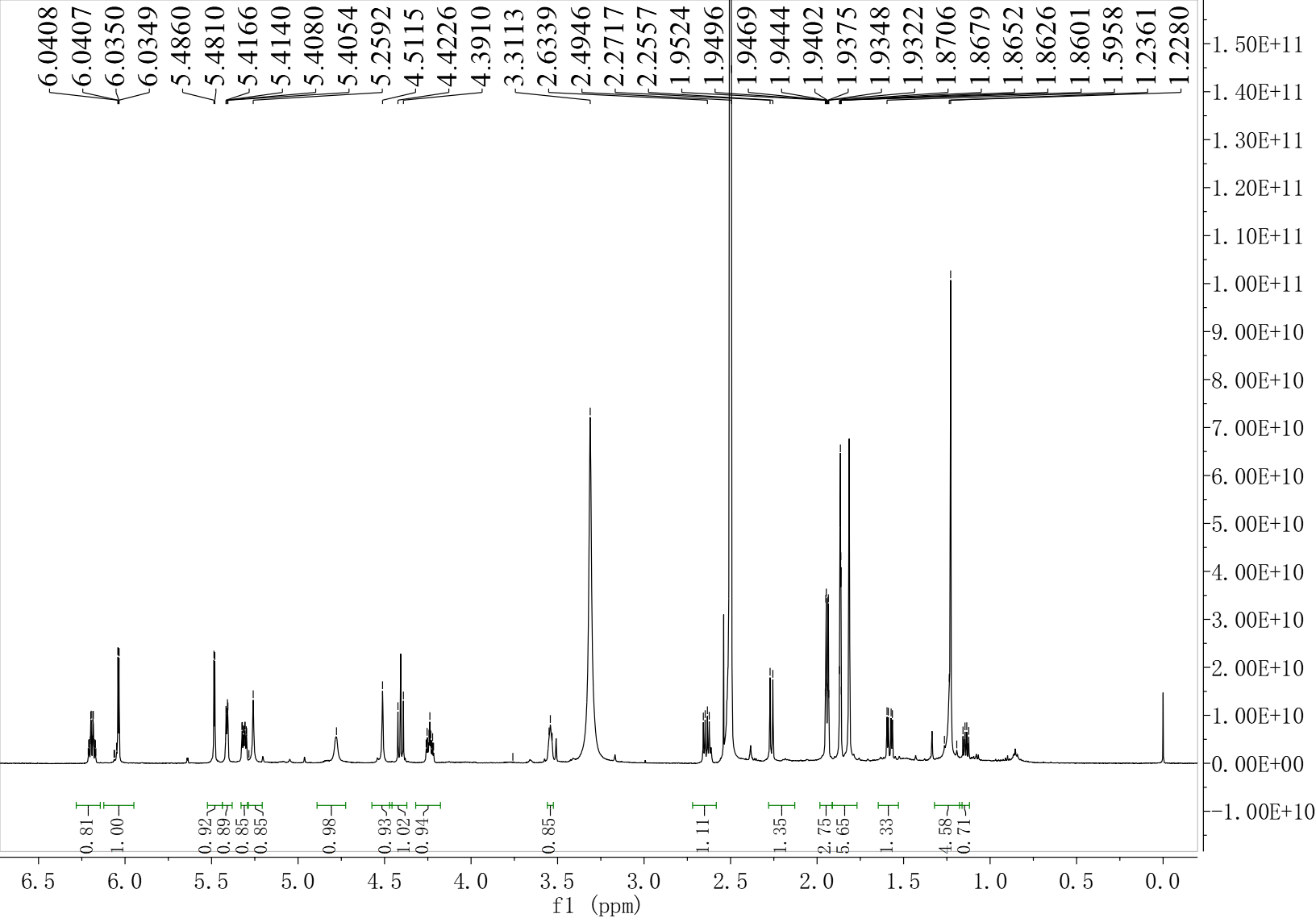


**Figure S4-3** ^1^H NMR (DMSO‑d_6_, 600 MHz) spectrum of compound **4**.


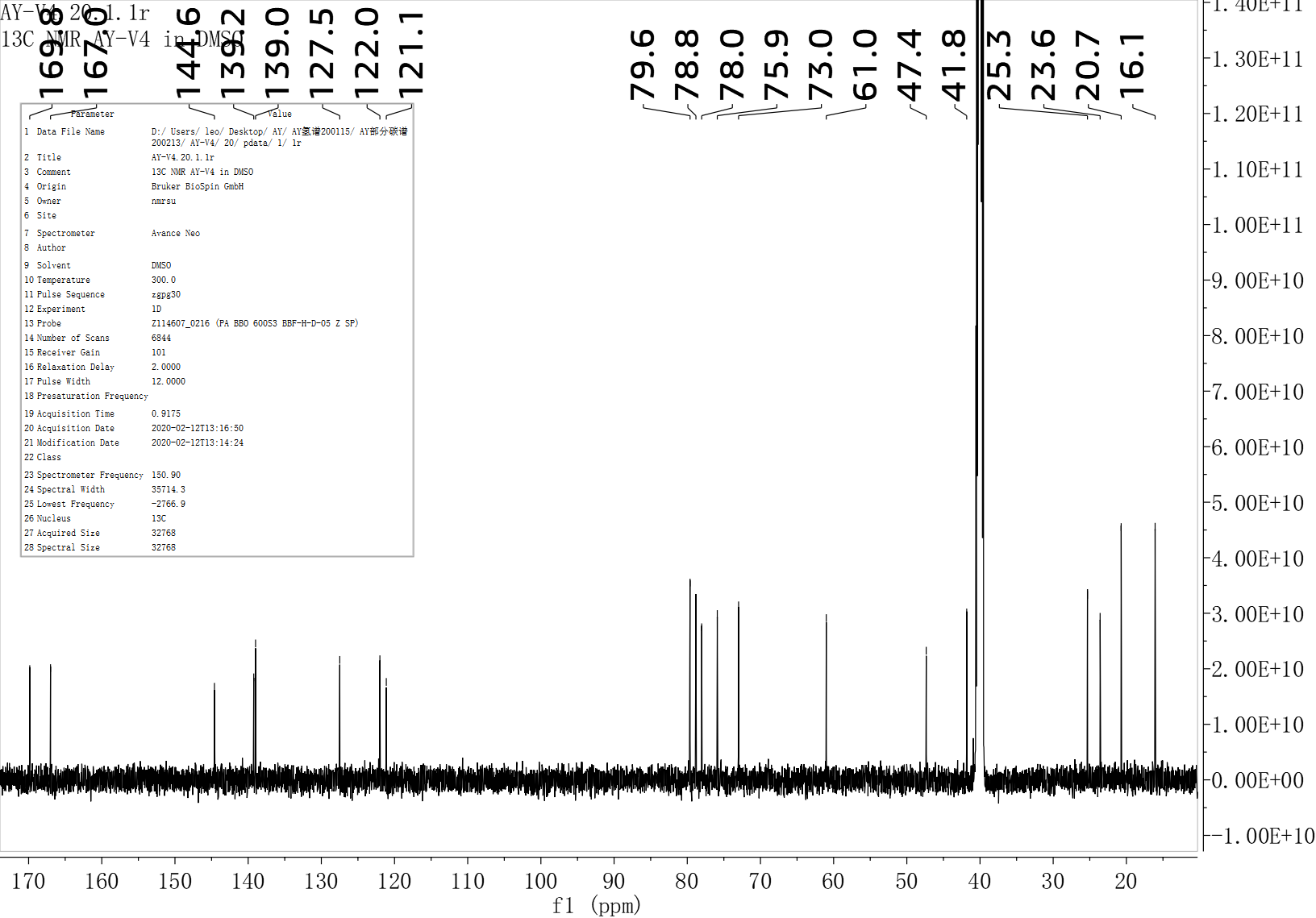


**Figure S4-4** ^13^C NMR (DMSO‑d_6_, 150 MHz) spectrum of compound **4**.


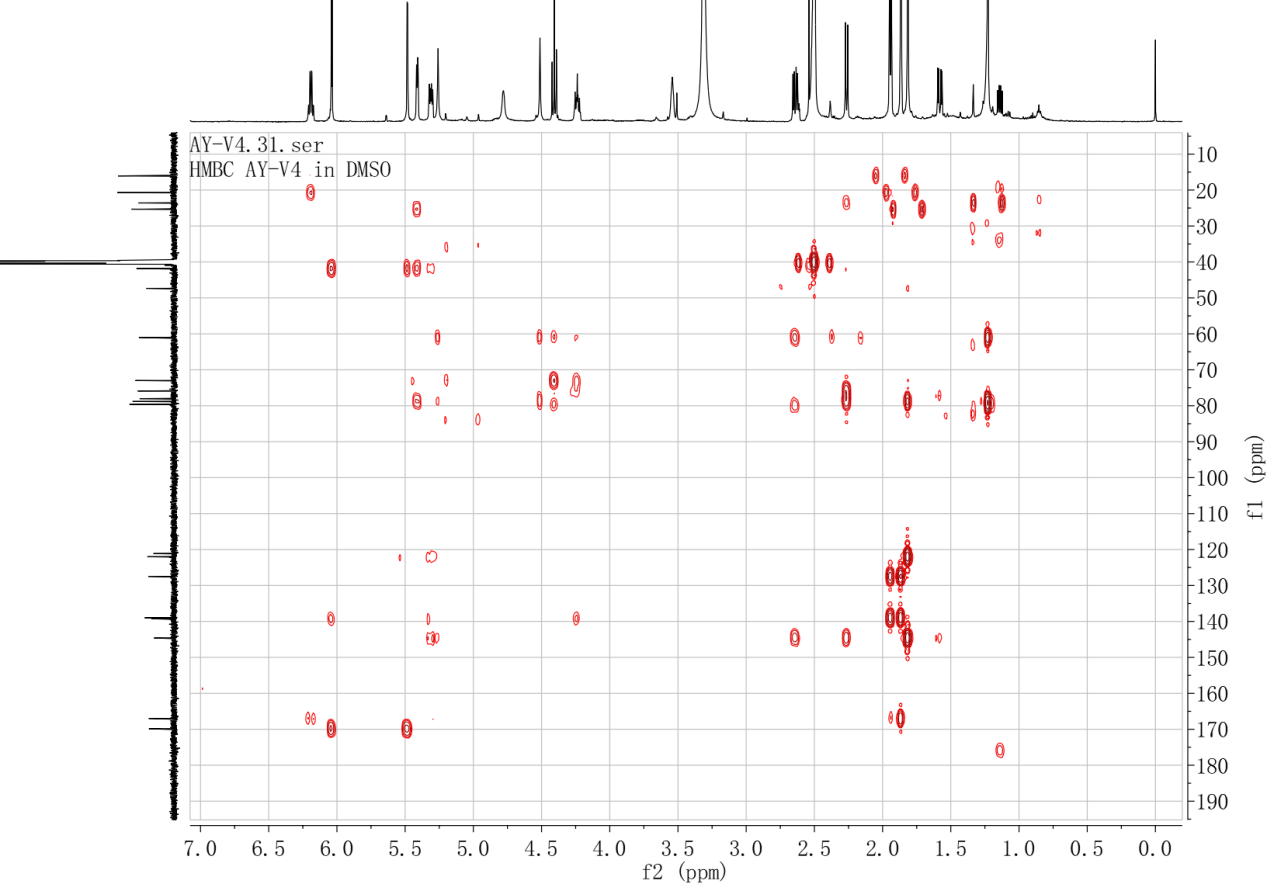


**Figure S4-5** HMBC (DMSO‑d_6_) spectrum of compound **4**.


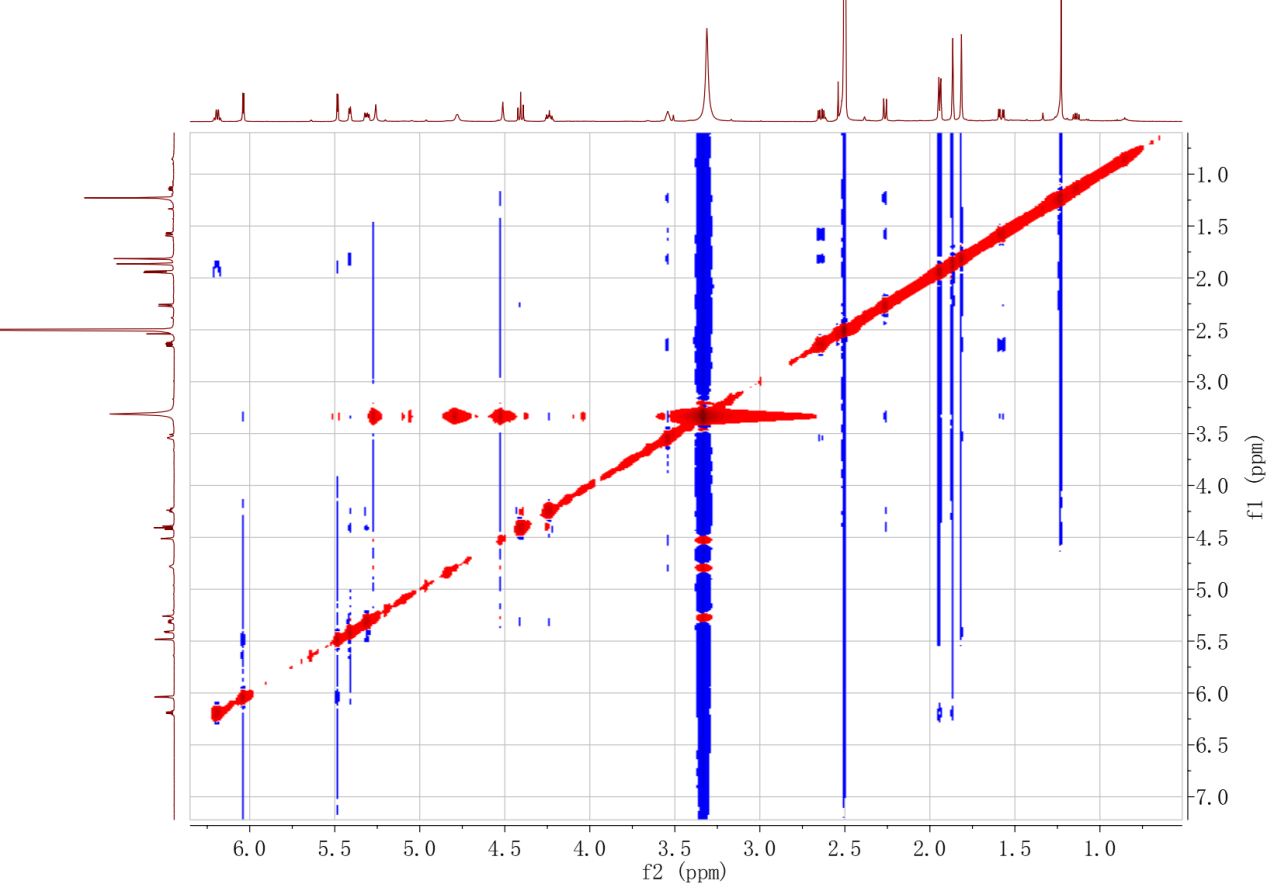


**Figure S4-6** NOESY (DMSO‑d_6_) spectrum of compound **4**.

PDB:1MOX


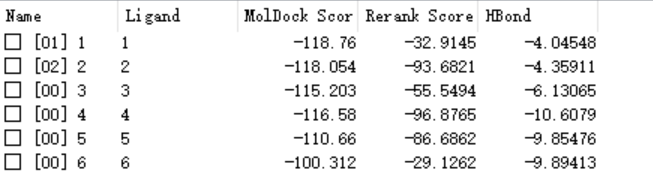


PDB:4MZV


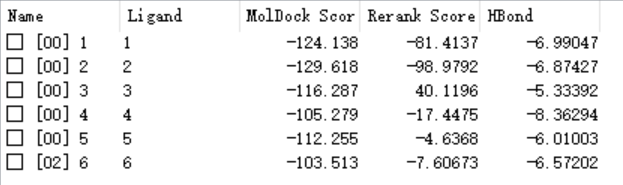


PDB:4RAO


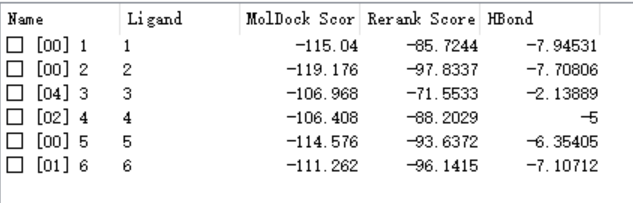


PDB:6AVZ


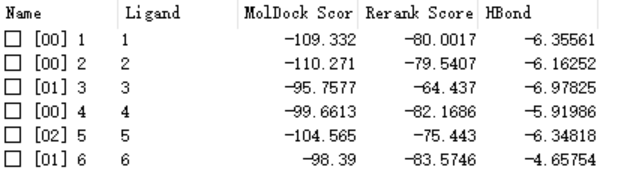


PDB:6M7X


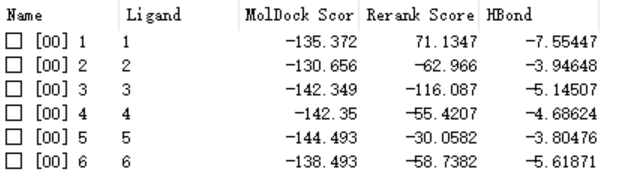


**Figure S5** Docking results of compound **1** to **6**
