# Supplementary material for: Guaianolide Sesquiterpenes With Significant Antiproliferative Activities From the Leaves of Artemisia argyi
Source: Front Chem. 2021 Jun 24;9:698700. doi: 10.3389/fchem.2021.698700 (PMC8263895; doi:10.3389/fchem.2021.698700)
Supplement: Supplementary file 4 [file DataSheet1.ZIP › CD/v4.pdf]

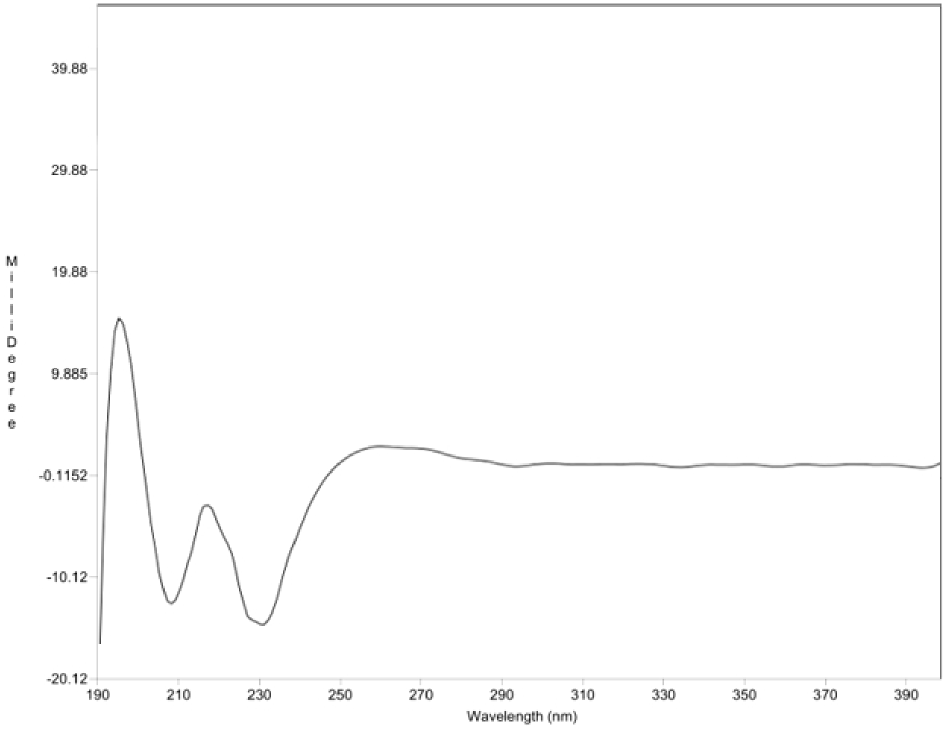


**Bio-Kine Software V4.74   Date : 2020-8-12   Time :  15:06:17**

**COMMENTS :**

File name : d:\孟大利\joe\2020-8-12\v4.bka
Savitzky-Golay Smooth of sav-golay
Window Points=15
Polynomial Order=3
Derivative=0
