# Supplementary material for: Guaianolide Sesquiterpenes With Significant Antiproliferative Activities From the Leaves of Artemisia argyi
Source: Front Chem. 2021 Jun 24;9:698700. doi: 10.3389/fchem.2021.698700 (PMC8263895; doi:10.3389/fchem.2021.698700)
Supplement: Supplementary file 6 [file DataSheet2.ZIP › compound1/20/pdata/1/email_MPI104276_10_1.pdf]

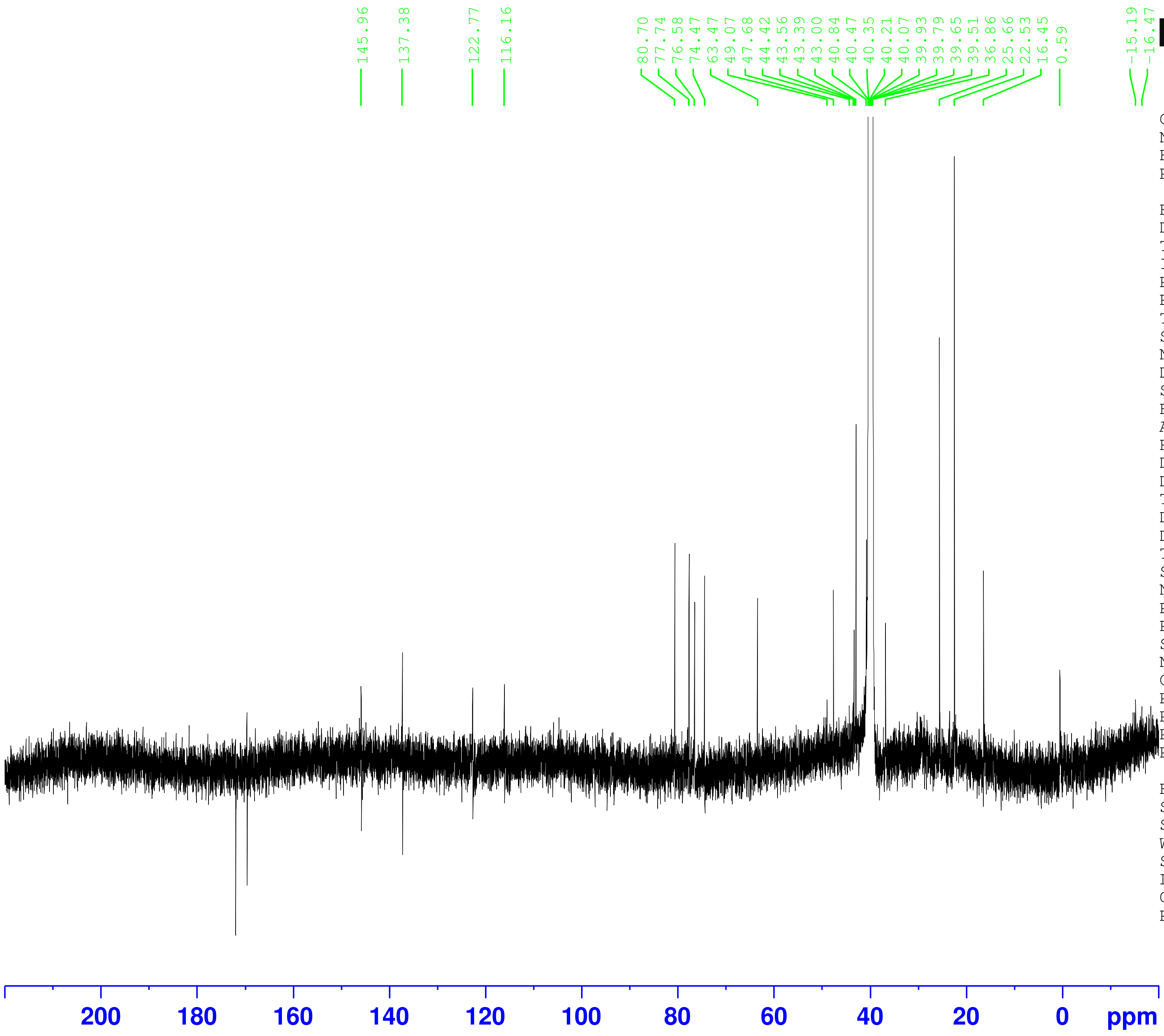

Current Data Parameters

NAME	MPI104276	
EXPNO	10	
PROCNO	1	

F2 - Acquisition Parameters

Date_	20200201	
Time	5.41	h
INSTRUM	spect	
PROBHD	Z132572_0018 (	
PULPROG	zgpg30	
TD	65536	
SOLVENT	DMSO	
NS	4096	
DS	4	
SWH	36231.883	Hz
FIDRES	0.552855	Hz
AQ	0.9043968	sec
RG	191.17	
DW	13.800	usec
DE	18.00	usec
TE	295.1	K
D1	2.00000000	sec
D11	0.03000000	sec
TD0	1	
SFO1	150.9178988	MHz
NUC1	13C	
P1	10.00	usec
PLW1	35.12699890	W
SFO2	600.1324005	MHz
NUC2	1H	
CPDPRG[2	waltz16	
PCPD2	80.00	usec
PLW2	15.90499973	W
PLW12	0.35786000	W
PLW13	0.17972000	W

F2 - Processing parameters

SI	32768	
SF	150.9028085	MHz
WDW	EM	
SSB	0	
LB	1.00	Hz
GB	0	
PC	1.40	
